# Primary Immunodeficiency Diseases: An Update on the Classification from the International Union of Immunological Societies Expert Committee for Primary Immunodeficiency

**DOI:** 10.3389/fimmu.2014.00162

**Published:** 2014-04-22

**Authors:** Waleed Al-Herz, Aziz Bousfiha, Jean-Laurent Casanova, Talal Chatila, Mary Ellen Conley, Charlotte Cunningham-Rundles, Amos Etzioni, Jose Luis Franco, H. Bobby Gaspar, Steven M. Holland, Christoph Klein, Shigeaki Nonoyama, Hans D. Ochs, Erik Oksenhendler, Capucine Picard, Jennifer M. Puck, Kate Sullivan, Mimi L. K. Tang

**Affiliations:** ^1^Department of Pediatrics, Kuwait University, Kuwait City, Kuwait; ^2^Allergy and Clinical Immunology Unit, Department of Pediatrics, Al-Sabah Hospital, Kuwait City, Kuwait; ^3^Clinical Immunology Unit, Casablanca Children’s Hospital, Ibn Rochd Medical School, King Hassan II University, Casablanca, Morocco; ^4^St. Giles Laboratory of Human Genetics of Infectious Diseases, Rockefeller Branch, The Rockefeller University, New York, NY, USA; ^5^Laboratory of Human Genetics of Infectious Diseases, Necker Branch, INSERM UMR1163, Imagine Institut, Necker Medical School, University Paris Descartes, Paris, France; ^6^Division of Immunology, Children’s Hospital Boston, Boston, MA, USA; ^7^Department of Medicine and Pediatrics, Mount Sinai School of Medicine, New York, NY, USA; ^8^Meyer Children’s Hospital-Technion, Haifa, Israel; ^9^Group of Primary Immunodeficiencies, University of Antioquia, Medellin, Colombia; ^10^UCL Institute of Child Health, London, UK; ^11^Laboratory of Clinical Infectious Diseases, National Institute of Allergy and Infectious Diseases, Bethesda, MD, USA; ^12^Dr. von Hauner Children’s Hospital, Ludwig-Maximilians-University Munich, Munich, Germany; ^13^Department of Pediatrics, National Defense Medical College, Saitama, Japan; ^14^Department of Pediatrics, Seattle Children’s Research Institute, University of Washington, Seattle, WA, USA; ^15^Department of Clinical Immunology, Hôpital Saint-Louis, Assistance Publique-Hôpitaux de Paris, Paris, France; ^16^Sorbonne Paris Cité, Université Paris Diderot, Paris, France; ^17^Centre d’Étude des Déficits Immunitaires (CEDI), Hôpital Necker-Enfants Malades, AP-HP, Paris, France; ^18^Department of Pediatrics, UCSF Benioff Children’s Hospital, University of California San Francisco, San Francisco, CA, USA; ^19^Department of Pediatrics, Division of Allergy Immunology, The Children’s Hospital of Philadelphia, Philadelphia, PA, USA; ^20^Murdoch Childrens Research Institute, Melbourne, VIC, Australia; ^21^Department of Paediatrics, University of Melbourne, Melbourne, VIC, Australia; ^22^Department of Allergy and Immunology, Royal Children’s Hospital, Melbourne, VIC, Australia

**Keywords:** primary immunodeficiencies, IUIS, classification, genetic defects, genotype

## Abstract

We report the updated classification of primary immunodeficiencies (PIDs) compiled by the Expert Committee of the International Union of Immunological Societies. In comparison to the previous version, more than 30 new gene defects are reported in this updated version. In addition, we have added a table of acquired defects that are phenocopies of PIDs. For each disorder, the key clinical and laboratory features are provided. This classification is the most up-to-date catalog of all known PIDs and acts as a current reference of the knowledge of these conditions and is an important aid for the molecular diagnosis of patients with these rare diseases.

## Background

The International Union of Immunological Societies (IUIS) Expert Committee on Primary Immunodeficiency met in New York on 19th–21st April 2013 to update the classification of human primary immunodeficiencies (PIDs). This report represents the most current and complete catalog of known PIDs. It serves as a reference for these conditions and provides a framework to help in the diagnostic approach to patients suspected to have PID.

As in previous reports, we have classified the conditions into major groups of PIDs and these are now represented in nine different tables. In each table, we list the condition, its genetic defect if known, and the major immunological and in some conditions the non-immunological abnormalities associated with the disease. The classification this year differs slightly from the previous edition in that Table 1 lists combined immunodeficiencies without non-immunologic phenotypes, whereas Table 2 refers to combined immunodeficiencies with syndromic features, as increasing numbers of these are being identified. The title and classification of Tables 3–8 present the same major PID groups as in the previous report.

**Table 1:** **Combined immunodeficiencies**.

Disease	Genetic defect/presumed pathogenesis	Inheritance	Circulating T cells	Circulating B cells	Serum Ig	Associated features	OMIM number
1. T^−^B^+^ severe combined immunodeficiency (SCID)							
(a) γc deficiency	Mutation of *IL-2RG*	XL	Markedly decreased	Normal or increased	Decreased	Markedly decreased NK cells	300400
	Defect in γ chain of receptors for IL-2, -4, -7, -9, -15, -21	
(b) JAK3 deficiency	Mutation of *JAK3*	AR	Markedly decreased	Normal or increased	Decreased	Markedly decreased NK cells	600173
	Defect in Janus-activating kinase 3	
(c) IL7Rα deficiency	Mutation of *IL7RA*	AR	Markedly decreased	Normal or increased	Decreased	Normal NK cells	146661
	Defect in IL-7 receptor α chain	
(d) CD45 deficiency^a^	Mutation of *PTPRC*	AR	Markedly decreased	Normal	Decreased	Normal γ/δ T cells	151460
	Defect in CD45	
(e) CD3δ deficiency	Mutation of *CD3D*	AR	Markedly decreased	Normal	Decreased	Normal NK cells	186790
	Defect in CD3δ chain of T cell antigen receptor complex					No γ/δ T cells	
(f) CD3ε deficiency^a^	Mutation of *CD3E*	AR	Markedly decreased	Normal	Decreased	Normal NK cells	186830
	Defect in CD3ε chain of T cell antigen receptor complex					No γ/δ T cells	
(g) CD3ζ deficiency^a^	Mutation of *CD3Z*	AR	Markedly decreased	Normal	Decreased	Normal NK cells	186740
	Defect in CD3ζ chain of T cell antigen receptor complex					No γ/δ T cells	
(h) Coronin-1A deficiency^a^	Mutation of *CORO1A* defective thymic egress of T cells and defective T cell locomotion	AR	Markedly decreased	Normal	Decreased	Detectable thymus EBV associated B cell lymphoproliferation	605000
2. T^−^B^−^SCID							
(i) DNA recombination defects							
(a) RAG 1 deficiency	Mutation of *RAG1*	AR	Markedly decreased	Markedly decreased	Decreased		601457
	Defective VDJ recombination; defect of recombinase activating gene (RAG) 1	
(a) RAG 2 deficiency	Mutation of *RAG2*	AR	Markedly decreased	Markedly decreased	Decreased		601457
	Defective VDJ recombination; defect of recombinase activating gene (RAG) 2	
(b) DCLRE1C (artemis) deficiency	Mutation of *ARTEMIS*	AR	Markedly decreased	Markedly decreased	Decreased	Radiation sensitivity	602450
	Defective VDJ recombination; defect in artemis DNA recombinase repair protein	
(c) DNA PKcs deficiency^a^	Mutation of *PRKDC*- Defective VDJ recombination; defect in DNA PKcs	AR	Markedly decreased	Markedly decreased	Decreased	Radiation sensitivity, microcephaly, and developmental defects	600899
	Recombinase repair protein	
(ii) Reticular dysgenesis, AK2 deficiency	Mutation of *AK2*	AR	Markedly decreased	Decreased or normal	Decreased	Granulocytopenia and deafness	103020
	Defective maturation of lymphoid and myeloid cells (stem cell defect)	
	Defect in mitochondrial adenylate kinase 2	
(iii) Adenosine deaminase (ADA) deficiency	Mutation of ADA absent *ADA* activity, elevated lymphotoxic metabolites (dATP, *S*-adenosyl homocysteine)	AR	Absent from birth (null mutations) or progressive decrease	Absent from birth of progressive decrease	Progressive decrease	Decreased NK cells, often with costochondral junction flaring, neurological features, hearing impairment, lung and liver manifestations; partial ADA deficiency may lead to delayed or milder presentation	102700
**Combined immunodeficiencies generally less profound than severe combined immunodeficiency**							
3. CD40 ligand deficiency	Mutation of *CD40LG* defects in CD40 ligand (CD40L; also called TNFSF5 or CD154) cause defective isotype switching and impaired dendritic cell signaling	XL	Normal; may progressively decrease	sIgM^+^ and sIgD^+^ B cells present, other surface isotype positive B cells absent	IgM increased or normal, other isotypes decreased	Neutropenia, thrombocytopenia; hemolytic anemia, biliary tract and liver disease, opportunistic infections	300386
4. CD40 deficiency^a^	Mutation of *CD40* (also called TNFRSF5) defects in CD40 cause defective isotype switching and impaired dendritic cell signaling	AR	Normal	IgM^+^ and IgD^+^ B cells present, other isotypes absent	IgM increased or normal, other isotypes decreased	Neutropenia, gastrointestinal and liver/biliary tract disease, opportunistic infections	109535
5. Purine nucleoside phosphorylase (PNP) deficiency	Mutation of *PNP*, absent PNP, and T cell and neurologic defects from elevated toxic metabolites, especially dGTP	AR	Progressive decrease	Normal	Normal or decreased	Autoimmune hemolytic anemia, neurological impairment	164050
6. CD3γ deficiency^a^	Mutation of *CD3G* defect in CD3 γ – component of the T cell antigen receptor complex	AR	Normal, but reduced TCR expression	Normal	Normal		186740
7. CD8 deficiency^a^	Mutation of *CD8A*, defects of CD8 α chain – important for maturation and function of CD8 T cells	AR	Absent CD8, normal CD4 cells	Normal	Normal		186910
8. ZAP70 deficiency	Mutation in ZAP70 intracellular signaling kinase, acts downstream of TCR	AR	Decreased CD8, normal CD4 cells	Normal	Normal	Autoimmunity in some cases	269840
9. MHC class I deficiency	Mutations in *TAP1, TAP2*, or *TAPBP* (tapasin) genes giving MHC class I deficiency	AR	Decreased CD8, normal CD4	Normal	Normal	Vasculitis; pyoderma gangrenosum	604571
10. MHC class II deficiency	Mutation in transcription factors for MHC class II proteins (*CIITA, RFX5, RFXAP, RFXANK* genes)	AR	Normal number, decreased CD4 cells	Normal	Normal or decreased	Failure to thrive, diarrhea, respiratory tract infections, liver/biliary tract disease	209920
11. ITK deficiency^a^	Mutations in *ITK* encoding IL-2-inducible T cell kinase required for TCR-mediated activation	AR	Progressive decrease	Normal	Normal or decreased	EBV-associated B cell lymphoproliferation, lymphoma	613011
						Normal or decreased IgG	
12. SH2D1A deficiency (XLP1)	Mutations in *SH2D1A* encoding an adaptor protein regulating intracellular signals	XL	Normal or increased activated T cells	Reduced memory B cells	Partially defective NK cell and CTL cytotoxic activity	Clinical and immunologic features triggered by EBV infection: HLH, lymphoproliferation, aplastic anemia, lymphoma	308240
						Hypogammaglobulinemia	
						Absent iNKT cells	
13. Cartilage hair hypoplasia	Mutations in *RMRP* (RNase MRP RNA) involved in processing of mitochondrial RNA and cell cycle control	AR	Varies from severely decreased (SCID) to normal; impaired lymphocyte proliferation	Normal	Normal or reduced. antibodies variably decreased	Can present just as combined immunodeficiency without other features of short-limbed dwarfism	250250
						Also see Table 2	
14. MAGT1 deficiency^a^	Mutations in *MAGT1*, impaired Mg^++^ flux leading to impaired TCR signaling	XL	Decreased CD4 cells reduced numbers of RTE, impaired T cell proliferation in response to CD3	Normal	Normal	EBV infection, lymphoma; viral infections, respiratory, and GI infections	300715
15. DOCK8 deficiency	Mutations in *DOCK8* – regulator of intracellular actin reorganization	AR	Decreased impaired T lymphocyte proliferation	Decreased, low CD27+ memory B cells	Low IgM, increased IgE	Low NK cells with impaired function, hypereosinophilia, recurrent infections; severe atopy, extensive cutaneous viral and bacterial (staph.) infections, susceptibility to cancer	243700
16. RhoH deficiency^a^	Mutations in *RHOH* – an atypical Rho GTPase transducing signals downstream of various membrane receptors	AR	Normal	Normal	Normal	HPV infection, lymphoma, lung granulomas, molluscum contagiosum	602037
			Low naïve T cells and RTE, restricted T cell repertoire and impaired T cells proliferation in response to CD3 stimulation	
17. MST1 deficiency	Mutations in *STK4* – a serine/threonine kinase	AR	Decreased/increased proportion of terminal differentiated effector memory cells (TEMRA), low naïve T cells, restricted T cell repertoire in the TEMRA population, and impaired T cells proliferation	Decreased	High	Recurrent bacterial, viral, and candidal infections; intermittent neutropenia; EBV-driven lymphoproliferation; lymphoma; congenital heart disease, autoimmune cytopenias; HPV infection	614868
18. TCRα deficiency^a^	Mutations in *TRAC* – essential component of the T cell receptor	AR	Normal all CD3 T cells expressed TCRγδ (or may be better to say: TCRαβ T cell deficiency), impaired T cells proliferation	Normal	Normal	Recurrent viral, bacterial, and fungal infections, immune dysregulation autoimmunity, and diarrhea	615387
19. LCK deficiency^a^	Defects in *LCK* – a proximal tyrosine kinase that interacts with TCR	AR	Normal total numbers but CD4+ T cell lymphopenia, low Treg numbers, restricted T cell repertoire, and impaired TCR signaling	Normal	Normal IgG and IgA and increased IgM	Diarrhea, recurrent infections, immune dysregulation autoimmunity	153390
20. MALT1 deficiency^a^	Mutations in *MALT1* – a caspase-like cysteine protease that is essential for nuclear factor kappa B activation	AR	Normal impaired T cells proliferation	Normal	Normal	Bacterial, fungal, and viral infections	604860
					Impaired antibody response	
21. IL-21R deficiency^a^	Defects in *IL-21R* – together with common gamma chain binds IL-21	AR	Abnormal T cell cytokine production; abnormal T cell proliferation to specific stimuli	Normal	Normal but impaired specific responses	Susceptibility to cryptosporidium and pneumocystis and cholangitis	605383
22. UNC119 deficiency^a^	Defects in *UNC119* – an activator of src tyrosine kinases	AD	Low T cells	Mostly low	Normal	Recurrent bacterial, fungal, and viral infections	604011
			CD4+ T cell lymphopenia, impaired TCR signaling	
23. CARD11 deficiency^a^	Defects in *CARD11* – acts as a scaffold for NF-κB activity in the adaptive immune response	AR	Normal predominance of naive T lymphocyte, impaired T cells proliferation	Normal predominance of transitional B lymphocytes	Absent/low	*Pneumocystis jiroveci* pneumonia, bacterial infections	615206
24. OX40 deficiency^a^	Defects in *OX40* – a co-stimulatory molecule expressed on activated T cells	AR	Normal T cell numbers	Normal B cell numbers	Normal	Kaposi’s sarcoma; impaired immunity to HHV8	615593
			Low levels of antigen-specific memory CD4+ cells	Lower frequency of memory B cells	
25. IKBKB deficiency^a^	Defects in *IKBKB* – encodes IkB kinase 2 a component of the NF-κB pathway	AR	Normal total T cells; absent regulatory and gd T cells; impaired TCR activation	Normal B cell numbers; impaired BCR activation	Decreased	Recurrent bacterial, viral, and fungal infections; clinical phenotype of SCID	615592
26. Activated PI3K-δ	Mutation in *PIK3CD*, PI3K-δ	AD gain-of-function	Decreased total numbers of T cells	Decreased total peripheral B cell and switched memory B cells; increased transitional B cells	Reduced IgG2 and impaired antibody to pneumococci and hemophilus	Respiratory infections, bronchiectasis; autoimmunity; chronic EBV, and CMV infection	602839
27. LRBA deficiency	Mutations in *LRBA* (lipopolysaccharide responsive beige-like anchor protein)	AR	Normal or decreased CD4 numbers; T cell dysregulation	Low or normal numbers of B cells	Reduced I IgG and IgA in most	Recurrent infections, inflammatory bowel disease, autoimmunity; EBV infections	606453
28. CD27 deficiency^a^	Mutations in *CD27*, encoding TNF-R member superfamily required for generation and long-term maintenance of T cell immunity	AR	Normal	No memory B cells	Hypogamma globulinemia following EBV infection	Clinical and immunologic features triggered by EBV infection, HLH	615122
						Aplastic anemia, lymphoma	
						Hypogammaglobulinemia	
						Low iNKT cells	
29. Omenn syndrome	Hypomorphic mutations in *RAG1, RAG2, artemis, IL7RA, RMRP, ADA, DNA ligase IV, IL-2RG, AK2*, or associated with DiGeorge syndrome; some cases have no defined gene mutation		Present; restricted T cell repertoire, and impaired function	Normal or decreased	Decreased, except increased IgE	Erythroderma, eosinophilia, adenopathies, hepatosplenomegaly	603554

*XL, X-linked inheritance; AR, autosomal recessive inheritance; AD, autosomal dominant inheritance; SCID, severe combined immune deficiencies; EBV, Epstein–Barr virus; Ca^++^, calcium; MHC, major histocompatibility complex, RTE, recent thymic emigrants, HPV, human papillomavirus*.

*^a^Ten or fewer unrelated cases reported in the literature*.

*Infants with SCID who have maternal T cells engraftment may have T cells that do not function normally; these cells may cause autoimmune cytopenias or graft versus host disease. Hypomorphic mutations in several of the genes that cause SCID may result in Omenn syndrome (OS), or “leaky” SCID or a less profound CID phenotype. Both OS and leaky SCID can be associated with higher numbers of T cells and reduced rather than absent activation responses when compared with typical SCID caused by null mutations. A spectrum of clinical findings including typical SCID, OS, leaky SCID, granulomas with T lymphopenia, autoimmunity, and CD4+ T lymphopenia can be found with RAG gene defects. RAC2 deficiency is a disorder of leukocyte motility and is reported in Table 5; however, one patient with RAC2 deficiency was found to have absent T cell receptor excision circles (TRECs) by newborn screening, but T cell numbers and mitogen responses were not impaired. For additional syndromic conditions with T cell lymphopenia, such as DNA repair defects, cartilage hair hypoplasia, IKAROS deficiency, and NEMO syndrome, see Tables 2 and 6; however, it should be noted that individuals with the most severe manifestations of these disorders could have clinical signs and symptoms of SCID. Severe folate deficiency (such as with malabsorption due to defects in folate carrier or transporter genes SLC10A1 or PCFT) and some metabolic disorders, such as methylmalonic aciduria, may present with reversible profound lymphopenia in addition to their characteristic presenting features*.

**Table 2:** **Combined immunodeficiencies with associated or syndromic features**.

Disease	Genetic defect/presumed pathogenesis	Inheritance	Circulating T cells	Circulating B cells	Serum Ig	Associated features	OMIM number
1. Congenital thrombocytopenia							
(a) Wiskott– Aldrich syndrome (WAS)	Mutations in *WAS*; cytoskeletal, and immunologic synapse defect affecting hematopoietic stem cell derivatives	XL	Progressive decrease, abnormal lymphocyte responses to anti-CD3	Normal	Decreased IgM: antibody to polysaccharides particularly decreased; often increased IgA and IgE	Thrombocytopenia with small platelets; eczema; lymphoma; autoimmune disease; IgA nephropathy; bacterial and viral infections. XL thrombocytopenia is a mild form of WAS, and XL neutropenia is caused by missense mutations in the GTPase binding domain of WASP	301000
(b) WIP deficiency^a^	Mutations in *WIPF1*; cytoskeletal and immunologic synapse defect affecting hematopoietic stem cell derivatives	AR	Reduced, defective lymphocyte responses to anti-CD3	Low	Normal, except for increased IgE	Recurrent infections; eczema; thrombocytopenia. WAS-*like* phenotype	614493
2. DNA repair defects (other than those in Table 1)							
(a) Ataxia–telangiectasia	Mutations in *ATM*; disorder of cell cycle checkpoint; and DNA double-strand break repair	AR	Progressive decrease	Normal	Often decreased IgA, IgE, and IgG subclasses; increased IgM monomers; antibodies variably decreased	Ataxia; telangiectasia; pulmonary infections; lymphoreticular and other malignancies; increased alpha fetoprotein and increased radiosensitivity; chromosomal instability	208900
(b) Ataxia–telangiectasia-like disease (ATLD)^a^	Hypomorphic mutations in *MRE11*; disorder of cell cycle checkpoint and DNA double-strand break repair	AR	Progressive decrease	Normal	Antibodies variably decreased	Moderate ataxia; pulmonary infections; severely increased radiosensitivity	604391
(c) Nijmegen breakage syndrome	Hypomorphic mutations in *NBS1 (Nibrin)*; disorder of cell cycle checkpoint and DNA double-strand break repair	AR	Progressive decrease	Variably reduced	Often decreased IgA, IgE, and IgG subclasses; increased IgM; antibodies variably decreased	Microcephaly; bird-like face; lymphomas; solid tumors; increased radiosensitivity; chromosomal instability	251260
(d) Bloom syndrome	Mutations in *BLM*; RecQ-like helicase	AR	Normal	Normal	Reduced	Short stature; bird-like face; sun-sensitive erythema; marrow failure; leukemia; lymphoma; chromosomal instability	210900
(e) Immunodeficiency with centromeric instability and facial anomalies (ICF)	Mutations in DNA methyltransferase *DNMT3B* (ICF1) resulting in defective DNA methylation	AR	Decreased or normal; responses to PHA may be decreased	Decreased or normal	Hypogamma globulinemia; variable antibody deficiency	Facial dysmorphic features; macroglossia; bacterial/opportunistic infections; malabsorption; cytopenias; malignancies; multiradial configurations of chromosomes 1, 9, 16; no DNA breaks	242860
(f) Immunodeficiency with centromeric instability and facial anomalies (ICF)	Mutations in *ZBTB24* (ICF2)	AR	Decreased or normal; responses to PHA may be decreased	Decreased or normal	Hypogamma globulinemia; variable antibody deficiency	Facial dysmorphic features; macroglossia; bacterial/opportunistic infections; malabsorption; cytopenias; malignancies; multiradial configurations of chromosomes 1, 9, 16	242860
(g) PMS2 deficiency	Mutations in *PMS2*, resulting in class switch recombination deficiency due to impaired mismatch repair	AR	Normal	Switched and non-switched B cells are reduced	Low IgG and IgA, elevated IgM, abnormal antibody responses	Recurrent infections; café-au-lait spots; lymphoma, colorectal carcinoma, brain tumor	600259
(h) RNF168 deficiency^a^	Mutations in *RNF168*, resulting in defective DNA double-strand break repair	AR	Normal	Normal	Low IgG or low IgA	Short stature; mild motor control to ataxia and normal intelligence to learning difficulties; mild facial dysmorphism to microcephaly; increased radiosensitivity	611943
(i) MCM4 deficiency	Mutations in *MCM4* (minichromosome maintenance complex component 4) gene involved in DNA replication and repair	AR	Normal	Normal	Normal	Viral infections (EBV, HSV, VZV) Adrenal failure Short stature	609981
3. Thymic defects with additional congenital anomalies							
(a) DiGeorge anomaly	Contiguous gene defect in 90% affecting thymic development; may also be due to heterozygous mutation in *TBX1* (chromosome 22q11.2 deletion or TBX1 haploinsufficient syndrome)	*De novo* defect (majority) or AD	Decreased or normal; 5% have <1500 CD3 T cells/μL	Normal	Normal or decreased	Hypoparathyroidism, conotruncal malformation; abnormal facies; large deletion (3 Mb) in 22q11.2 (or rarely a deletion in 10p)	188400
(b) CHARGE syndrome	Variable defects of the thymus and associated T cell abnormalities often due to deletions or mutations in *CHD7, SEMA3E*, or as yet unknown genes	*De novo* defect (majority) or AD	Decreased or normal; some have <1500 CD3 T cells/μL	Normal	Normal or decreased	Coloboma, heart anomaly, choanal atresia, retardation, genital and ear anomalies	214800 608892
4. Immune-osseous dysplasias							
(a) Cartilage hair hypoplasia	Mutations in *RMRP* (RNase MRP RNA) involved in processing of mitochondrial RNA and cell cycle control	AR	Varies from severely decreased (SCID) to normal; impaired lymphocyte proliferation	Normal	Normal or reduced. Antibodies variably decreased	Short-limbed dwarfism with metaphyseal dysostosis, sparse hair, bone marrow failure, autoimmunity, susceptibility to lymphoma and other cancers, impaired spermatogenesis, neuronal dysplasia of the intestine	250250
(b) Schimke syndrome	Mutations in *SMARCAL1* involved in chromatin remodeling	AR	Decreased	Normal	Normal	Short stature, spondiloepiphyseal dysplasia, intrauterine growth retardation, nephropathy; bacterial, viral, and fungal infections; may present as SCID; bone marrow failure	242900
5. Hyper-IgE syndromes (HIES)							
(a) AD-HIES (Job’s syndrome)	Dominant-negative heterozygous mutations in *STAT3*	AD Often *de novo* defect	Normal Th-17 and T follicular helper cells decreased	Normal Switched and non-switched memory B cells are reduced; BAFF level increased	Elevated IgE; specific antibody production decreased	Distinctive facial features (broad nasal bridge), eczema, osteoporosis, and fractures, scoliosis, delay of shedding primary teeth, hyperextensible joints, bacterial infections (skin and pulmonary abscesses, pneumatoceles) due to *Staphylococcus aureus*, candidiasis, aneurysm formation	147060
(i) Tyk2 deficiency^a^	Mutation in *TYK2*	AR	Normal, but multiple cytokine signaling defect	Normal	(±) Elevated IgE	Susceptibility to intracellular bacteria (*Mycobacteria, Salmonella*), fungi, and viruses	611521
(ii) DOCK8 deficiency	Mutations in *DOCK8* – regulator of intracellular actin reorganization	AR	Decreased impaired T lymphocyte proliferation	Decreased, low CD27+ memory B cells	Low IgM, increased IgE	Low NK cells with impaired function, hypereosinophilia, recurrent infections; severe atopy, extensive cutaneous viral and bacterial (staph.) infections, susceptibility to cancer	243700
6. Dyskeratosis congenital (DKC)							
(a) XL-DKC	Mutations in dyskerin *(DKC1)* (Hoyeraal–Hreidarsson syndrome)	XL	Progressive decrease	Progressive decrease	Variable	Intrauterine growth retardation, microcephaly, nail dystrophy, recurrent infections, digestive tract involvement, pancytopenia, reduced number and function of NK cells	305000
(b) AR-DKC due to NHP2 deficiency	Mutation in *NOLA2 (NHP2)*	AR	Decreased	Variable	Variable	Pancytopenia, sparse scalp hair and eyelashes, prominent periorbital telangiectasia, and hypoplastic/dysplastic nails	613987
(c) AR-DKC due to NOP10 deficiency	Mutation in *NOLA3 (NOP10 PCFT)*	AR	Decreased	Variable	Variable	Pancytopenia, sparse scalp hair and eyelashes, prominent periorbital telangiectasia, and hypoplastic/dysplastic nails	224230
(d) AR-DKC due to RTEL1 deficiency	Mutation in *(RTEL1)*	AR	Decreased	Variable	Variable	Pancytopenia, sparse scalp hair and eyelashes, prominent periorbital telangiectasia, and hypoplastic/dysplastic nails	608833
(e) AD-DKC due to TERC deficiency	Mutation in *TERC*	AD	Variable	Variable	Variable	Reticular hyperpigmentation of the skin, dystrophic nails, osteoporosis premalignant leukokeratosis of the mouth mucosa, palmar hyperkeratosis, anemia, pancytopenia	127550
(f) AD-DKC due to TERT deficiency	Mutation in *TERT*	AD	Variable	Variable	Variable	Reticular hyperpigmentation of the skin, dystrophic nails, osteoporosis premalignant leukokeratosis of the mouth mucosa, palmar hyperkeratosis, anemia, pancytopenia	614742
(g) AD-DKC due to TINF2 deficiency	Mutation in *TINF2*	AD	Variable	Variable	Variable	Reticular hyperpigmentation of the skin, dystrophic nails, osteoporosis premalignant leukokeratosis of the mouth mucosa, palmar hyperkeratosis, anemia, pancytopenia	613990
7. Defects of vitamin B12 and folate metabolism							
(a) TCN2 deficiency	Mutation in *TCN2*; encodes transcobalamin, a transporter of cobalamin into blood cells	AR	Normal	Variable	Decreased	Megaloblastic anemia, pancytopenia, untreated for prolonged periods results in mental retardation	275350
(b) SLC46A1 deficiency	Mutation in *SLC46A1*; a proton coupled folate transporter	AR	Variable numbers and activation profile	Variable	Decreased	Megaloblastic anemia, failure to thrive untreated for prolonged periods results in mental retardation	229050
(c) MTHFD1^a^ deficiency	Mutations in *MTHFD1*; essential for processing of single-carbon folate derivatives	AR	Low	Low	Decreased	Megaloblastic anemia, failure to thrive neutropenia, seizures, mental retardation	
8. Comel–Netherton syndrome	Mutations in *SPINK5* resulting in lack of the serine protease inhibitor LEKTI, expressed in epithelial cells	AR	Normal	Switched and non-switched B cells are reduced	Elevated IgE and IgA	Congenital ichthyosis, bamboo hair, atopic diathesis, increased bacterial infections, failure to thrive	256500
					Antibody variably decreased	
9. Winged helix deficiency (Nude)^a^	Defects in forkhead box N1 transcription factor encoded by *FOXN1*	AR	Markedly decreased	Normal	Decreased	Alopecia, abnormal thymic epithelium, impaired T cell maturation	600838
10. ORAI-I deficiency^a^	Mutation in *ORAI1*, a Ca^++^ release-activated channel (CRAC) modulatory component	AR	Normal number, but defective TCR-mediated activation	Normal	Normal	Autoimmunity, anhydrotic ectodermic dysplasia, non-progressive myopathy defective TCR-mediated activation	610277
11. STIM1 deficiency^a^	Mutations in *STIM1*, a stromal interaction molecule 1	AR	Normal number, but defective TCR-mediated activation	Normal	Normal	Autoimmunity, anhydrotic ectodermal dysplasia, non-progressive myopathy defective TCR-mediated activation	605921
12. STAT5b deficiency^a^	Mutations in *STAT5B*, signal transducer, and transcription factor, essential for normal signaling from IL-2 and 15, key growth factors for T and NK cells	AR	Modestly decreased	Normal	Normal	Growth-hormone insensitive dwarfism	245590
						Dysmorphic features	
						Eczema	
						Lymphocytic interstitial pneumonitis, autoimmunity	
13. Hepatic veno-occlusive disease with immunodeficiency (VODI)	Mutations in *SP110*	AR	Normal (decreased memory T cells)	Normal (decreased memory B cells)	Decreased IgG, IgA, IgM, absent germinal centers, absent tissue plasma cells	Hepatic veno-occlusive disease; *Pneumocystis jiroveci* pneumonia; susceptibility to CMV, *Candida*; thrombocytopenia; hepatosplenomegaly	235550
14. IKAROS deficiency^a^	Mutation in *IKAROS*	AD *de novo*	Normal, but impaired lymphocyte proliferation	Absent	Presumably decreased	Anemia, neutropenia, thrombocytopenia	Not assigned
15. FILS syndrome^a^	Mutation in *POLE1*; defective DNA replication	AR	Low naïve T cells; decreased T cell proliferation	Low memory B cells	Decreased IgM and IgG; lack of antibodies to polysaccharide antigens	Mild facial dysmorphism (malar hypoplasia, high forehead), livedo, short stature; recurrent upper and lower respiratory tract infections, recurrent pulmonary infections, and recurrent meningitis	615139
16. Immunodeficiency with multiple intestinal atresias	Mutation in *TTC7A* [tetratricopeptide repeat (TPR) domain 7A] protein of unknown function	AR	Variable, but sometimes absent	Normal	Decreased	Multiple intestinal atresias, often with intrauterine polyhydramnios and early demise; some with SCID phenotype	243150

*SCID, severe combined immune deficiencies; XL, X-linked inheritance; AR, autosomal recessive inheritance; AD, autosomal dominant inheritance; MSMD, Mendelian susceptibility of mycobacterial disease*.

*^a^Ten or fewer unrelated cases reported in the literature*.

*T and B cell number and function in these disorders exhibit a wide range of abnormality; the most severely affected cases meet diagnostic criteria for SCID or leaky SCID and require immune system restoring therapy such as allogeneic hematopoietic cell transplantation. While not all DOCK8-deficient patients have elevated serum IgE, most have recurrent viral infections and malignancies as a result of combined immunodeficiency. AR-HIES due to Tyk2 deficiency is also listed in Table 6, because of its association with atypical mycobacterial disease resulting in MSMD. Riddle syndrome is caused by mutations in a gene involved in DNA double-strand break repair and is associated with hypogammaglobulinemia. Autosomal dominant and autosomal recessive forms of dyskeratosis congenita are included in this table. IKAROS-deficiency represents a single prematurely born infant who died at the age of 87 days and who had absent B and NK cells and non-functional T cells*.

**Table 3:** **Predominantly antibody deficiencies**.

Disease	Genetic defect/presumed pathogenesis	Inheritance	Serum Ig	Associated features	OMIM number
1. Severe reduction in all serum immunoglobulin isotypes with profoundly decreased or absent B cells					
(a) BTK deficiency	Mutations in *BTK*, a cytoplasmic tyrosine kinase activated by crosslinking of the BCR	XL	All isotypes decreased in majority of patients; some patients have detectable immunoglobulins	Severe bacterial infections; normal numbers of pro-B cells	300300
(b) μ Heavy chain deficiency	Mutations in μ heavy chain; essential component of the pre-BCR	AR	All isotypes decreased	Severe bacterial infections; normal numbers of pro-B cells	147020
(c) λ5 Deficiency^a^	Mutations in l5; part of the surrogate light chain in the pre-BCR	AR	All isotypes decreased	Severe bacterial infections; normal numbers of pro-B cells	146770
(d) Igα deficiency^a^	Mutations in Iga *(CD79a)*; part of the pre-BCR and BCR	AR	All isotypes decreased	Severe bacterial infections; normal numbers of pro-B cells	112205
(e) Igβ deficiency^a^	Mutations in Igb *(CD79β)*; part of the pre-BCR and BCR	AR	All isotypes decreased	Severe bacterial infections; normal numbers of pro-B cells	147245
(f) BLNK deficiency^a^	Mutations in *BLNK*; a scaffold protein that binds to BTK	AR	All isotypes decreased	Severe bacterial infections; normal numbers of pro-B cells	604615
(g) PI3 kinase deficiency^a^	Mutations in *PIK3R1*; a kinase involved in signal transduction in multiple cell types	AR	All isotypes decreased	Severe bacterial infections; decreased or absent pro-B cells	171833
(h) E47 transcription factor deficiency^a^	Mutations in *TCF3*; a transcription factor required for control of B cell development	AD	All isotypes decreased	Recurrent bacterial infections	147141
(i) Myelodysplasia with hypogammaglobulinemia	May have monosomy 7, trisomy 8, or dyskeratosis congenita	Variable	One or more isotypes may be decreased	Infections; decreased number of pro-B cells	Not assigned
(j) Thymoma with immunodeficiency	Unknown	None	One or more isotypes may be decreased	Bacterial and opportunistic infections; autoimmunity; decreased number of pro-B cells	Not assigned
2. Severe reduction in at least two serum immunoglobulin isotypes with normal or low number of B cells					
(a) Common variable immunodeficiency disorders	Unknown	Variable	Low IgG and IgA and/or IgM	Clinical phenotypes vary: most have recurrent infections, some have polyclonal lymphoproliferation, autoimmune cytopenias, and/or granulomatous disease	Not assigned
(b) ICOS deficiency^a^	Mutations in *ICOS*; a co-stimulatory molecule expressed on T cells	AR	Low IgG and IgA and/or IgM	Recurrent infections; autoimmunity, gastroenteritis, granuloma in some	604558
(c) CD19 deficiency^a^	Mutations in *CD19*; transmembrane protein that amplifies signal through BCR	AR	Low IgG and IgA and/or IgM	Recurrent infections; may have glomerulonephritis	107265
(d) CD81 deficiency^a^	Mutations in *CD81*; transmembrane protein that amplifies signal through BCR	AR	Low IgG, low or normal IgA and IgM	Recurrent infections; may have glomerulonephritis	186845
(e) CD20 deficiency^a^	Mutations in *CD20*; a B cell surface receptor involved in B cell development and plasma cell differentiation	AR	Low IgG, normal or elevated IgM and IgA	Recurrent infections	112210
(f) CD21 deficiency^a^	Mutations in *CD21*; also known as complement receptor 2 and forms part of the CD19 complex	AR	Low IgG; impaired anti-pneumococcal response	Recurrent infections	614699
(g) TACI deficiency	Mutations in *TNFRSF13B* (TACI); a TNF receptor family member found on B cells and is a receptor for BAFF and APRIL	AD or AR or complex	Low IgG and IgA and/or IgM	Variable clinical expression	604907
(h) LRBA deficiency	Mutations in *LRBA* (lipopolysaccharide responsive beige-like anchor protein)	AR	Reduced I IgG and IgA in most	Recurrent infections, inflammatory bowel disease, autoimmunity; EBV infections	606453
(i) BAFF receptor deficiency^a^	Mutations in *TNFRSF13C* (BAFF-R); a TNF receptor family member found on B cells and is a receptor for BAFF	AR	Low IgG and IgM	Variable clinical expression	606269
(j) TWEAK^a^	Mutations in *TWEAK*	AD	Low IgM and IgA; lack of anti-pneumococcal antibody	Pneumonia, bacterial infections, warts; thrombocytopenia. neutropenia	602695
(k) NFKB2 deficiency^a^	Mutations in *NFKB2*; an essential component of the non-canonical NF-κB pathway	AD	Low IgG and IgA and IgM	Recurrent infections	615577
(l) Warts, hypogammaglobulinemia, infections, myelokathexis (WHIM) syndrome	Gain-of-function mutations of *CXCR4*, the receptor for CXCL12	AD	Panhypogammaglobulinemia, decreased B cells	Warts/human papilloma virus (HPV) infection Neutropenia Reduced B cell number Hypogammaglobulinemia	193670
3. Severe reduction in serum IgG and IgA with normal/elevated IgM and normal numbers of B cells					
(a) CD40L deficiency	Mutations in *CD40LG* (also called *TNFSF5* or *CD154*)	XL	IgG and IgA decreased; IgM may be normal or increased; B cell numbers may be normal or increased	Bacterial and opportunistic infections, neutropenia, autoimmune disease	300386
(b) CD40 deficiency^a^	Mutations in *CD40* (also called *TNFRSF5*)	AR	Low IgG and IgA; normal or raised IgM	Bacterial and opportunistic infections, neutropenia, autoimmune disease	109535
(c) AID deficiency	Mutations in *AICDA* gene	AR	IgG and IgA decreased; IgM increased	Bacterial infections, enlarged lymph nodes, and germinal centers	605257
(d) UNG deficiency	Mutations in *UNG*	AR	IgG and IgA decreased; IgM increased	Enlarged lymph nodes and germinal centers	191525
4. Isotype or light chain deficiencies with generally normal numbers of B cells					
(a) Ig heavy chain mutations and deletions	Mutation or chromosomal deletion at 14q32	AR	One or more IgG and/or IgA subclasses as well as IgE may be absent	May be asymptomatic	Not assigned
(b) κ Chain deficiency^a^	Mutations in Kappa constant gene	AR	All immunoglobulins have lambda light chain	Asymptomatic	147200
(c) Isolated IgG subclass deficiency	Unknown	Variable	Reduction in one or more IgG subclass	Usually asymptomatic; a minority may have poor antibody response to specific antigens and recurrent viral/bacterial infections	Not assigned
(d) IgA with IgG subclass deficiency	Unknown	Variable	Reduced IgA with decrease in one or more IgG subclass	Recurrent bacterial infections	Not assigned
(e) PRKC δ deficiency^a^	Mutation in *PRKCD*; encoding a member of the protein kinase C family critical for regulation of cell survival, proliferation, and apoptosis	AR	Low IgG levels; IgA and IgM above the normal range	Recurrent infections; EBV chronic infection Lymphoproliferation SLE-like autoimmunity (nephrotic and antiphospholipid syndromes)	615559
(f) Activated PI3K-δ	Mutation in *PIK3CD*, PI3K-δ	AD gain-of-function	Reduced IgG2 and impaired antibody to pneumococci and hemophilus	Respiratory infections, bronchiectasis; autoimmunity; chronic EBV, CMV infection	602839
(g) Selective IgA deficiency	Unknown	Variable	IgA decreased/absent	Usually asymptomatic; may have recurrent infections with poor antibody responses to carbohydrate antigens; may have allergies or autoimmune disease. A very few cases progress to CVID, others coexist with CVID in the family	137100
5. Specific antibody deficiency with normal Ig concen-trations and normal numbers of B cells	Unknown	Variable	Normal	Reduced ability to produce antibodies to specific antigens	Not assigned
6. Transient hypogammaglobulinemia of infancy with normal numbers of B cells	Unknown	Variable	IgG and IgA decreased	Normal ability to produce antibodies to vaccine antigens, usually not associated with significant infections	Not assigned

*XL, X-linked inheritance; AR, autosomal recessive inheritance; AD, autosomal dominant inheritance; BTK, Bruton tyrosine kinase; BLNK, B cell linker protein; AID, activation-induced cytidine deaminase; UNG, uracil-DNA glycosylase; ICOS, inducible costimulator; Ig(κ), immunoglobulin or κ light chain type*.

*^a^Ten or fewer unrelated cases reported in the literature*.

*Several autosomal recessive disorders that might previously have been called CVID have been added to Table 3. CD81 is normally co-expressed with CD19 on the surface of B cells. As for CD19 mutations, mutations in CD81 result in normal numbers of peripheral blood B cells, low serum IgG, and an increased incidence of glomerulonephritis. Single patient with a homozygous mutation in CD20 and CD21 has been reported*.

*Common variable immunodeficiency disorders (CVID) include several clinical and laboratory phenotypes that may be caused by distinct genetic and/or environmental factors. Some patients with CVID and no known genetic defect have markedly reduced numbers of B cells as well as hypogammaglobulinemia. Alterations in TNFRSF13B (TACI) and TNFRSF13C (BAFF-R) sequences may represent disease-modifying mutations rather than disease causing mutations. CD40L and CD40 deficiency are included in Table 1 as well as this table. A small minority of patients with XLP (Table 4), WHIM syndrome (Table 6), ICF (Table 2), VOD1 (Table 2), thymoma with immunodeficiency (Good syndrome), or myelodysplasia are first seen by an immunologist because of recurrent infections, hypogammaglobulinemia, and normal or reduced numbers of B cells. Patients with GATA2 mutations (Table 5) may have markedly reduced numbers of B cells, as well as decreased monocytes and NK cells, and a predisposition to myelodysplasia but they do not usually have an antibody deficiency*.

**Table 4:** **Diseases of immune dysregulation**.

Disease	Genetic defect/presumed pathogenesis	Inheritance	Circulating T cells	Circulating B cells	Functional defect	Associated features	OMIM number
1. Familial hemophagocytic lymphohistiocytosis (FHL) syndromes							
1.1 FHL syndromes without hypopigmentation							
(a) Perforin deficiency (FHL2)	Mutations in *PRF1*; perforin is a major cytolytic protein	AR	Increased activated T cells	Normal	Decreased to absent NK and CTL activities (cytotoxicity)	Fever, hepatosplenomegaly (HSMG), hemophagocytic lymphohistiocytosis (HLH), cytopenias	603553
(b) UNC13D/Munc13-4 deficiency (FHL3)	Mutations in *UNC13D ^a^*; required to prime vesicles for fusion	AR	Increased activated T cells	Normal	Decreased to absent NK and CTL activities (cytotoxicity and/or degranulation)	Fever, HSMG, HLH, cytopenias	608898
(c) Syntaxin 11 deficiency (FHL4)	Mutations in *STX11*, required for secretory vesicle fusion with the cell membrane	AR	Increased activated T cells	Normal	Decreased NK activity (cytotoxicity and/or degranulation)	Fever, HSMG, HLH, cytopenias	603552
(d) STXBP2/Munc18-2 deficiency (FHL5)	Mutations in *STXBP2*, required for secretory vesicle fusion with the cell membrane	AR	Increased activated T cells	Normal	Decreased NK and CTL activities (cytotoxicity and/or degranulation)	Fever, HSMG, HLH, cytopenias	613101
1.2. FHL syndromes with hypopigmentation							
(a) Chediak–Higashi syndrome	Mutations in *LYST* Impaired lysosomal trafficking	AR	Increased activated T cells	Normal	Decreased NK and CTL activities (cytotoxicity and/or degranulation)	Partial albinism Recurrent infections, fever HSMG, HLH Giant lysosomes, neutropenia, cytopenias Bleeding tendency Progressive neurological dysfunction	214500
(b) Griscelli syndrome, type 2	Mutations in *RAB27A* encoding a GTPase that promotes docking of secretory vesicles to the cell membrane	AR	Normal	Normal	Decreased NK and CTL activities (cytotoxicity and/or degranulation)	Partial albinism, fever, HSMG, HLH, cytopenias	607624
(c) Hermansky–Pudlak syndrome, type 2	Mutations in *AP3B1* gene, encoding for the b subunit of the AP-3 complex	AR	Normal	Normal	Decreased NK and CTL activities (cytotoxicity and/or degranulation)	Partial albinism Recurrent infections Pulmonary fibrosis Increased bleeding Neutropenia HLH	608233
2. Lymphoproliferative syndromes							
(a) SH2D1A deficiency (XLP1)	Mutations in *SH2D1A* encoding an adaptor protein regulating intracellular signaling	XL	Normal or increased activated T cells	Reduced memory B cells	Partially defective NK cell and CTL cytotoxic activity	Clinical and immunological features triggered by EBV infection: HLH Lymphoproliferation, aplastic anemia, lymphoma Hypogammaglobulinemia Absent iNKT cells	308240
(b) XIAP deficiency (XLP2)	Mutations in *XIAP/BIRC4* encoding an inhibitor of apoptosis	XL	Normal or increased activated T cells; low/normal iNK T cells	Normal or reduced memory B cells	Increased T cells susceptibility to apoptosis to CD95 and enhanced activation-induced cell death (AICD)	EBV infection, splenomegaly, lymphoproliferation HLH, colitis, IBD, hepatitis Low iNKT cells	300635
(c) ITK deficiency^a^	Mutations in *ITK* encoding IL-2 inducible T cell kinase required for TCR-mediated activation	AR	Progressive decrease	Normal	Decreased T cell activations	EBV-associated B cell lymphoproliferation, lymphoma Normal or decreased IgG	613011
(d) CD27 deficiency^a^	Mutations in *CD27*, encoding TNF-R member superfamily required for generation and long-term maintenance of T cell immunity	AR	Normal	No memory B cells	Low T and NK cells functions	Clinical and immunological features triggered by EBV infection: HLH Aplastic anemia, lymphoma, hypogammaglobulinemia Low iNKT cells	615122
3. Genetic defects of regulatory T cells							
(a) IPEX, immune dysregulation, polyendocrinopathy, enteropathy X-linked	Mutations in *FOXP3*, encoding a T cell transcription factor	XL	Normal	Normal	Lack of (and/or impaired function of) CD4^+^ CD25^+^ FOXP3^+^ regulatory T cells (Tregs)	Autoimmune enteropathy Early-onset diabetes Thyroiditis, hemolytic anemia, thrombocytopenia, eczema Elevated IgE, IgA	304790
(b) CD25 deficiency^a^	Mutations in *IL-2RA*, encoding IL-2Rα chain	AR	Normal to decreased	Normal	No CD4+ C25+ cells with impaired function of Tregs cells	Lymphoproliferation, autoimmunity. Impaired T cell proliferation	606367
(c) STAT5b deficiency^a^	Mutations in *STAT5B*, signal transducer, and transcription factor, essential for normal signaling from IL-2 and 15, key growth factors for T and NK cells	AR	Modestly decreased	Normal	Impaired development and function of γδT cells, Tregs, and NK cells Low T cell proliferation	Growth-hormone insensitive dwarfism	245590
						Dysmorphic features	
						Eczema	
						Lymphocytic interstitial pneumonitis, autoimmunity	
4. Autoimmunity without lymphoproliferation							
(a) APECED (APS-1), autoimmune polyendocrinopathy with candidiasis and ectodermal dystrophy	Mutations in *AIRE*, encoding a transcription regulator needed to establish thymic self-tolerance	AR	Normal	Normal	AIRE-1 serves as checkpoint in the thymus for negative selection of autoreactive T cells and for generation of Tregs	Autoimmunity: hypoparathyroidism hypothyroidism, adrenal insufficiency, diabetes, gonadal dysfunction, and other endocrine abnormalities	240300
						Chronic mucocutaneous candidiasis	
						Dental enamel hypoplasia	
						Alopecia areata	
						Enteropathy, pernicious anemia	
(b) ITCH deficiency^a^	Mutations in *ITCH*, an E3 ubiquitin ligase catalyzes the transfer of ubiquitin to a signaling protein in the cell including phospholipase Cγ1 (PLCγ1)	AR	Not assessed	Not assessed	Itch deficiency may cause immune dysregulation by affecting both anergy induction in autoreactive effector T cells and generation of Tregs	Early-onset chronic lung disease (interstitial pneumonitis)	613385
						Autoimmune disorder (thyroiditis, type I diabetes, chronic diarrhea/enteropathy, and hepatitis)	
						Failure to thrive, developmental delay, dysmorphic facial features	
5. Autoimmune lymphoproliferative syndrome (ALPS)							
(a) ALPS–FAS	Germinal mutations in *TNFRSF6*, encoding CD95/Fas cell surface apoptosis receptor^b^	AD	Increased CD4^−^CD8^−^ TCRα/β double negative (DN) T cells	Normal, low memory B cells	Apoptosis defect FAS mediated	Splenomegaly, adenopathies, autoimmune cytopenias	601859
		AR^c^				Increased lymphoma risk	
						IgG and A normal or increased	
						Elevated FasL and IL-10, vitamin B12	
(b) ALPS– FASLG	Mutations in *TNFSF6*, Fas ligand for CD95 apoptosis	AR	Increased DN T cells	Normal	Apoptosis defect FAS mediated	Splenomegaly, adenopathies, autoimmune cytopenias, SLE	134638
						Soluble FasL is not elevated	
(c) ALPS–caspase 10^a^	Mutations in *CASP10*, intracellular apoptosis pathway	AD	Increased DN T cells	Normal	Defective lymphocyte apoptosis	Adenopathies, splenomegaly, autoimmunity	603909
(d) ALPS–caspase 8^a^	Mutations in *CASP8*, intracellular apoptosis, and activation pathways	AR	Slightly increased DN T cells	Normal	Defective lymphocyte apoptosis and activation	Adenopathies, splenomegaly, bacterial and viral infections, hypogammaglobulinemia	607271
(e) FADD deficiency^a^	Mutations in *FADD* encoding an adaptor molecule interacting with FAS, and promoting apoptosis	AR	Increased DN T cells	Normal	Defective lymphocyte apoptosis	Functional hyposplenism, bacterial and viral infections	613759
						Recurrent episodes of encephalopathy and liver dysfunction	
(f) CARD11 gain-of-function (GOF) mutations^a^	GOF mutations in *CARD11*, encoding a protein required for antigen receptor–induced NF-κB activation in B and T lymphocytes	AD	Normal	Increased M^+^D^+^CD19^+^ CD20^+^ B cells	Constitutive activation of NF-κB in B & T	Lymphoproliferation	606445
						Bacterial and viral infections	
						EBV chronic infection	
						Autoimmune cytopenia	
						Hypogammaglobulinemia	
(g) PRKCδ deficiency^a^	Mutations in *PRKCD*, encoding a member of the protein kinase C family critical for regulation of cell survival, proliferation, and apoptosis	AR	Normal	Low memory B cells and elevation of CD5 B cells	Apoptotic defect in B cells	Recurrent infections; EBV chronic infection	615559
						Lymphoproliferation	
						SLE-like autoimmunity (nephrotic and antiphospholipid syndromes)	
						HypoIgG	
6. Immune dysregulation with colitis							
(a) IL-10 deficiency^a^	Mutations in *IL-10*, encoding IL-10	AR	Normal	Normal	No functional IL-10 secretion	Inflammatory bowel disease (IBD) folliculitis	Not assigned
						Recurrent respiratory diseases	
						Arthritis	
(b) IL-10Rα deficiency	Mutations in *IL-10RA*, encoding IL-10R1	AR	Normal	Normal	Leukocytes, no response to IL-10	IBD, folliculitis	613148
						Recurrent respiratory diseases	
						Arthritis, lymphoma	
(c) IL-10Rβ deficiency	Mutations in *IL-10RB*, encoding IL-10R2	AR	Normal	Normal	Leukocytes, no response to IL-10, IL-22, IL-26, IL-28A, IL-28B, and IL-29	IBD, folliculitis	612567
						Recurrent respiratory diseases	
						Arthritis, lymphoma	
7. Type 1 interferonopathies							
(a) TREX1 deficiency, Aicardi–Goutieres syndrome 1 (AGS1)	Mutations in *TREX1*, encoding nuclease involves in clearing cellular nucleic debris	AR	Not assessed	Not assessed	Intracellular accumulation of abnormal single-stranded (ss) DNA species leading to increased CSF alpha-IFN production	Progressive encephalopathy intracranial calcifications	606609
		AD^e^				Cerebral atrophy, leukodystrophy	
						HSMG, thrombocytopenia	
						Elevated hepatic transaminases	
						Chronic cerebrospinal fluid (CSF) lymphocytosis	
(b) RNASEH2B deficiency, AGS2	Mutations in *RNASEH2B*, encoding nuclease subunit involves in clearing cellular nucleic debris	AR	Not assessed	Not assessed	Intracellular accumulation of abnormal ss-DNA species leading to increased CSF alpha-IFN production	Progressive encephalopathy intracranial calcifications	610326
						Cerebral atrophy, leukodystrophy	
						HSMG, thrombocytopenia	
						Elevated hepatic transaminases	
						Chronic CSF lymphocytosis	
(c) RNASEH2C deficiency, AGS3	Mutations in *RNASEH2C*, encoding nuclease subunit involves in clearing cellular nucleic debris	AR	Not assessed	Not assessed	Intracellular accumulation of abnormal ss-DNA species leading to increased CSF alpha-IFN production	Progressive encephalopathy intracranial calcifications	610330
						Cerebral atrophy, leukodystrophy	
						HSMG, thrombocytopenia	
						Elevated hepatic transaminases	
						Chronic CSF lymphocytosis	
(d) RNASEH2A deficiency, AGS4^a^	Mutations in *RNASEH2A*, encoding nuclease subunit involves in clearing cellular nucleic debris	AR	Not assessed	Not assessed	Intracellular accumulation of abnormal ss-DNA species leading to increased CSF alpha-IFN production	Progressive encephalopathy intracranial calcifications	606034
						Cerebral atrophy, leukodystrophy	
						HSMG, thrombocytopenia	
						Elevated hepatic transaminases	
						Chronic CSF lymphocytosis	
(e) SAMHD1 deficiency, AGS5	Mutations in *SAMHD1*, encoding negative regulator of the immunostimulatory DNA response	AR	Not assessed	Not assessed	Induction of the cell intrinsic antiviral response, apoptosis, and mitochondrial DNA destruction leading to increased CSF alpha-IFN production	Progressive encephalopathy intracranial calcifications	612952
						Cerebral atrophy, leukodystrophy	
						HSMG, thrombocytopenia, anemia elevated lactates	
						Chronic CSF lymphocytosis	
						Skin vasculitis, mouth ulcers, arthropathy	
(f) ADAR1 deficiency, AGS6	Mutations in *ADAR1*, encoding an RNA-specific adenosine deaminase	AR	Not assessed	Not assessed	Catalyzes the deamination of adenosine to inosine in dsRNA substrates markedly elevated CSF IFN-alpha	Progressive encephalopathy intracranial calcification Severe developmental delay, leukodystrophy	615010
(g) Spondyloenchondro-dysplasia with immune dysregulation (SPENCD)	Mutations in *ACP5*, encoding tartrate-resistant acid phosphatase (TRAP)	AR	Not assessed	Not assessed	Upregulation of IFN-alpha and type I IFN-stimulated genes	Recurrent bacterial and viral infections, intracranial calcification	607944
						SLE-like autoimmunity (Sjögren’s syndrome, hypothyroidism, inflammatory myositis, Raynaud’s disease and vitiligo), hemolytic anemia, thrombocytopenia, skeletal dysplasia, short stature	

*XL, X-linked inheritance; AR, autosomal recessive inheritance; AD, autosomal dominant inheritance; FHL, familial hemophagocytic lymphohistiocytosis; HLH, hemophagocytic lymphohistiocytosis; HSMG, hepatosplenomegaly; DN, double negative; SLE, systemic lupus erythematous; IBD, inflammatory bowel disease; CSF, chronic cerebrospinal fluid*.

*^a^Ten or fewer unrelated cases reported in the literature*.

*^b^Somatic mutations of TNFRSF6 cause a similar phenotype (ALPS–sFAS), see Table 9. Germinal mutation and somatic mutation of TNFRSF6 can be associated in some ALPS–FAS patients*.

*^c^AR ALPS–FAS patients have a most severe clinical phenotype*.

*^d^Somatic mutations in KRAS or NRAS can give this clinical phenotype associated autoimmune leukoproliferative disease (RALD) and are now included in Table 9 entitled phenocopies of PID*.

*^e^*De novo* dominant TREX1 mutations have been reported*.

*Fourteen new disorders have been added to Table 4. Two new entries have been added in the table, including immune dysregulation with colitis and Type 1 interferonopathies. EBV-driven lymphoproliferation is also observed in MAGT1 deficiency (Table 1)*.

**Table 5:** **Congenital defects of phagocyte number, function, or both**.

Disease	Genetic defect/presumed pathogenesis	Inheritance	Affected cells	Affected function	Associated features	OMIM number
1. Defects of neutrophil function						
(a) Severe congenital neutropenia 1 (ELANE deficiency)	Mutation in *ELANE*: misfolded protein response, increased apoptosis	AD	N	Myeloid differentiation	Susceptibility to MDS/leukemia	202700
(b) SCN2^a^ (GFI 1 deficiency)	Mutation in *GFI1*: loss of repression of ELANE	AD	N	Myeloid differentiation	B/T lymphopenia	613107
(c) SCN3 (Kostmann disease)	Mutation in *HAX1*: control of apoptosis	AR	N	Myeloid differentiation	Cognitive and neurological defects in patients with defects in both HAX1 isoforms, susceptibility to MDS/leukemia	610738
(d) SCN4 (G6PC3 deficiency)	Mutation in *G6PC3*: abolished enzymatic activity of glucose-6-phosphatase, aberrant glycosylation, and enhanced apoptosis of N and F	AR	N + F	Myeloid differentiation, chemotaxis, O_2_^−^production	Structural heart defects, urogenital abnormalities, inner ear deafness, and venous angiectasias of trunks and limbs	612541
(e) SCN5	Mutation in *VPS45 controls vesicular trafficking*	AR	N + F	Myeloid differentiation, migration	Extramedullary hematopoiesis, bone marrow fibrosis, nephromegaly	615285
(f) Glycogen storage disease type 1b	Mutation in *G6PT1*: glucose-6-phosphate transporter 1	AR	N + M	Myeloid differentiation, chemotaxis, O_2_^−^production	Fasting hypoglycemia, lactic acidosis, hyperlipidemia, hepatomegaly	232220
(g) Cyclic neutropenia	Mutation in *ELANE*: misfolded protein response	AD	N	Differentiation	Oscillations of other leukocytes and platelets	162800
(h) X-linked neutropenia/^a^myelodysplasia	Mutation in *WAS*: regulator of actin cytoskeleton (loss of auto-inhibition)	XL, gain-of-function	N + M	Mitosis	Monocytopenia	300299
(i) P14/LAMTOR2 deficiency^a^	Mutation in *ROBLD3/LAMTOR2*: endosomal adaptor protein 14	AR	N + L Mel	Endosome biogenesis	Neutropenia	610389
					Hypogammaglobulinemia	
					↓CD8 cytotoxicity	
					Partial albinism	
					Growth failure	
(j) Barth syndrome	Mutation in tafazzin *(TAZ)* gene: abnormal lipid structure of mitochondrial membrane, defective carnitine metabolism	XL	N	Myeloid differentiation	Cardiomyopathy, myopathy, growth retardation	302060
(k) Cohen syndrome	Mutation in *COH1* gene: Pg unknown	AR	N	Myeloid differentiation	Retinopathy, developmental delay, facial dysmorphisms	216550
(l) Clericuzio syndrome poikiloderma with neutropenia	Mutation in *C16ORF57*, affects genomic integrity	AR	N	Myeloid differentiation	Poikiloderma, neutropenia, MDS	613276
2. Defects of motility						
(a) Leukocyte adhesion deficiency type 1 (LAD1)	Mutation in *ITGB2*: adhesion protein (CD18)	AR	N + M + L + NK	Adherence, chemotaxis, endocytosis, T/NK cytotoxicity	Delayed cord separation, skin ulcers Periodontitis Leukocytosis	116920
(b) Leukocyte adhesion deficiency type 2 (LAD2)^a^	Mutation in *FUCT1*: GDP-fucose transporter	AR	N + M	Rolling, chemotaxis	Mild LAD type 1 features plus hh-blood group plus mental and growth retardation	266265
(c) Leukocyte adhesion deficiency type 3 (LAD3)	Mutation in *KINDLIN3*: Rap1-activation of β1–3 integrins	AR	N + M + L + NK	Adherence, chemotaxis	LAD type 1 plus bleeding tendency	612840
(d) Rac 2 deficiency^a^	Mutation in *RAC2*: regulation of actin cytoskeleton	AD	N	Adherence, chemotaxis, O_2_^−^production	Poor wound healing, leukocytosis	602049
(e) β-Actin deficiency^a^	Mutation in *ACTB*: cytoplasmic actin	AD	N + M	Motility	Mental retardation, short stature	102630
(f) Localized juvenile periodontitis	Mutation in *FPR1*: chemokine receptor	AR	N	Formylpeptide induced chemotaxis	Periodontitis only	136537
(g) Papillon–Lefèvre syndrome	Mutation in *CTSC*: cathepsin C activation of serine proteases	AR	N + M	Chemotaxis	Periodontitis, palmoplantar hyperkeratosis in some patients	245000
(h) Specific granule deficiency^a^	Mutation in *C/EBPE*: myeloid transcription factor	AR	N	Chemotaxis	Neutrophils with bilobed nuclei; absent secondary granules and defensins	245480
(i) Shwachman–Diamond syndrome	Mutation in *SBDS*: defective ribosome synthesis	AR	N	Chemotaxis	Pancytopenia, exocrine pancreatic insufficiency, chondrodysplasia	260400
3. Defects of respiratory burst						
(a) X-linked chronic granulomatous disease (CGD)	Mutation in *CYBB*: electron transport protein (gp91phox)	XL	N + M	Killing (faulty O_2_^−^production)	Recurrent bacterial infection, susceptibility to fungal infection, inflammatory gut manifestations McLeod phenotype in patients with deletions extending into the contiguous Kell locus	306400
(b) Autosomal recessive CGD – p22 phox deficiency	Mutation in *CYBA*: electron transport protein (p22phox)	AR	N + M	Killing (faulty O_2_^−^production)	Recurrent bacterial infection, susceptibility to fungal infection, and inflammatory gut manifestations	233690
(c) Autosomal recessive CGD – p47 phox deficiency	Mutation in *NCF1*: adapter protein (p47phox)	AR	N + M	Killing (faulty O_2_^−^production)	Recurrent bacterial infection, susceptibility to fungal infection, and inflammatory gut manifestations	233700
(d) Autosomal recessive CGD – p67 phox deficiency	Mutation in *NCF2*: activating protein (p67phox)	AR	N + M	Killing (faulty O_2_^−^production)	Recurrent bacterial infection, susceptibility to fungal infection, inflammatory gut manifestations	233710
(e) Autosomal recessive CGD – p40 phox deficiency^a^	Mutation in *NCF4*: activating protein (p40phox)	AR	N + M	Killing (faulty O_2_^−^production)	Inflammatory gut manifestations only	601488
4. Mendelian susceptibility to mycobacterial disease (MSMD)						
(a) IL-12 and IL-23 receptor β1 chain deficiency	Mutation in *IL-12RB1*: IL-12 and IL-23 receptor β1 chain	AR	L + NK	IFN-γ secretion	Susceptibility to *Mycobacteria* and *Salmonella*	209950
(b) IL-12p40 deficiency	Mutation in *IL-12B*: subunit p40 of IL-12/IL-23	AR	M	IFN-γ secretion	Susceptibility to *Mycobacteria* and *Salmonella*	161561
(c) IFN-γ receptor 1 deficiency	Mutation in *IFNGR1*: IFN-γR ligand binding chain	AR, AD	M + L	IFN-γ binding and signaling	Susceptibility to *Mycobacteria* and *Salmonella*	107470
(d) IFN-γ receptor 2 deficiency	Mutation in *IFNGR2*: IFN-γR accessory chain	AR	M + L	IFN-γ signaling	Susceptibility to *Mycobacteria* and *Salmonella*	147569
(e) STAT1 deficiency (AD form)^a^	Mutation in *STAT1* (loss of function)	AD	M + L	IFN-γ signaling	Susceptibility to *Mycobacteria*	600555
(f) Macrophage gp91 phox deficiency^a^	Mutation in *CYBB*: electron transport protein (gp 91 phox)	XL	Mf only	Killing (faulty O_2_^−^production)	Isolated susceptibility to *Mycobacteria*	306400
(g) IRF8-deficiency (AD form)^a^	Mutation in *IRF8*: IL-12 production by CD1c^+^ MDC	AD	CD1c+ MDC	Differentiation of CD1c+ MDC subgroup	Susceptibility to *Mycobacteria*	601565
(h) ISG15	Mutation in *ISG15*; an interferon (IFN) α/β-inducible, ubiquitin-like intracellular protein	AR	M + N + L	IFN-γ secretion	Susceptibility to *Mycobacteria*	14751
5. Other defects						
(a) IRF 8-deficiency (AR form)^a^	Mutation in *IRF8*: IL-12 production	AR	Monocytes peripheral DC	Cytopenias	Susceptibility to *Mycobacteria, Candida*, myeloproliferation	614893
(b) GATA2 deficiency (Mono MAC syndrome)	Mutation in *GATA2*: loss of stem cells	AD	Monocytes peripheral DC + NK + B	Multilineage cytopenias	Susceptibility to *Mycobacteria*, papilloma viruses, histoplasmosis, alveolar proteinosis, MDS/AML/CMML	137295
(c) Pulmonary alveolar proteinosis^a^	Mutation in *CSF2RA*	Biallelic mutations in pseudo-autosomal gene	Alveolar macro-phages	GM-CSF signaling	Alveolar proteinosis	306250

*XL, X-linked inheritance; AR, autosomal recessive inheritance; AD, autosomal dominant inheritance; ACTB, actin beta; B, B lymphocytes; CEBPE, CCAAT/enhancer-binding protein epsilon; CMML, chronic myelomonocytic leukemia; CTSC, cathepsin C; CYBA, cytochrome *b* alpha subunit; CYBB, cytochrome *b* beta subunit; DC, dendritic cells; ELANE, elastase neutrophil-expressed; GATA2, GATA binding protein 2; IFN, interferon; IFNGR1, interferon-gamma receptor subunit 1; IFNGR2, interferon-gamma receptor subunit 2; IL-12B, interleukin-12 beta subunit; IL-12RB1, interleukin-12 receptor beta 1; IFR8, interferon regulatory factor 8; F, fibroblasts; FPR1, formylpeptide receptor 1; FUCT1, fucose transporter 1; GFI1, growth factor independent 1; HAX1, HLCS1-associated protein X1; ITGB2, integrin beta-2; L, lymphocytes; M, monocytes–macrophages; MDC, myeloid dendritic cells; MDS, myelodysplasia; Mel, melanocytes; Mϕ, macrophages; MSMD, Mendelian susceptibility to mycobacterial disease; N, neutrophils; NCF1, neutrophil cytosolic factor 1; NCF2, neutrophil cytosolic factor 2; NCF4, neutrophil cytosolic factor 4; NK, natural killer cells; ROBLD3: roadblock domain containing 3; SBDS, Shwachman–Bodian–Diamond syndrome; STAT, signal transducer and activator of transcription*.

*^a^Ten or fewer unrelated cases reported in the literature*.

*Table 5 includes seven newly described genetic defects of phagocyte number and/or function including Barth syndrome, Cohen syndrome, and poikiloderma with neutropenia. In these three clinically well-known diseases, the genetic defects have been elucidated, although their molecular pathogenesis remains ill-defined. A new cause of autosomal recessive chronic granulomatous disease, namely a deficiency of the cytosolic activating protein p40 phox, has now been found in two CGD patients and is included under defects of respiratory burst. Under the heading of Mendelian susceptibility of mycobacterial disease (MSMD), two new entities were added: (a) a subgroup of X-linked gp91 phox deficiency with isolated susceptibility to mycobacteria and a defect of the respiratory burst in macrophages only; (b) an autosomal dominant form of IRF8-deficiency, resulting from a lack of CD1c+ myeloid dendritic cells that would normally secrete IL-12. The clinical phenotype of MSMD may vary, depending on the nature of the genetic defect. Finally, GATA2 deficiency was recently identified as the cause of the Mono MAC syndrome, with multilineage cytopenias (of monocytes, peripheral dendritic cells, NK- and B-lymphocytes) resulting in opportunistic infections (including mycobacteria), alveolar proteinosis, and malignancy*.

**Table 6:** **Defects in innate immunity**.

Disease	Genetic defect/presumed pathogenesis	Inheritance	Affected cell	Functional defect	Associated features	OMIM number
1. Anhidrotic ectodermal dysplasia with immunodeficiency (EDA-ID)						
(a) EDA-ID, X-linked (NEMO deficiency)	Mutations of *NEMO (IKBKG)*, a modulator of NF-κB activation	XL	Lymphocytes + monocytes	NF-κB signaling pathway	Various infections (bacteria, *Mycobacteria*, viruses, and fungi)	300248
					Colitis	
					EDA (not in all patients)	
					Hypogammaglobulinemia to specific antibody polysaccharides deficiency	
(b) EDA-ID, autosomal-dominant^a^	Gain-of-function mutations of *IKBA*, resulting in impaired activation of NF-κB	AD	Lymphocytes + monocytes	NF-κB signaling pathway	Various infections (bacteria, viruses, and fungi)	612132
					EDA	
					T cell defect	
2. TIR signaling pathway deficiency						
(a) IRAK-4 deficiency	Mutations of *IRAK-4*, a component of TLR- and IL-1R-signaling pathway	AR	Lymphocytes + granulocytes + monocytes	TIR–IRAK signaling pathway	Bacterial infections (pyogenes)	607676
(b) MyD88 deficiency	Mutations of *MYD88*, a component of the TLR and IL-1R signaling pathway	AR	Lymphocytes + granulocytes + monocytes	TIR–MyD88 signaling pathway	Bacterial infections (pyogenes)	612260
3. HOIL1 deficiency^a^	Mutation of *HOIL1*, a component of LUBAC	AR	Lymphocytes + granulocytes + monocytes	NF-κB signaling pathway	Bacterial infections (pyogenes)	Not assigned
					Autoinflammation	
					Amylopectinosis	
4. WHIM (Warts, hypogammaglobulinemia, infections, myelokathexis) syndrome	Gain-of-function mutations of *CXCR4*, the receptor for CXCL12	AD	Granulocytes + lymphocytes	Increased response of the CXCR4 chemokine receptor to its ligand CXCL12 (SDF-1)	Warts/human papilloma virus (HPV) infection	193670
					Neutropenia	
					Reduced B cell number	
					Hypogammaglobulinemia	
5. Epidermodysplasia verruciformis						
EVER1 deficiency	Mutations of *EVER1*	AR	Keratinocytes and leukocytes	EVER proteins may be involved in the regulation of cellular zinc homeostasis in lymphocytes	HPV (group B1) infections and cancer of the skin (typical EV)	226400
EVER2 deficiency	Mutations of *EVER2*	AR	Keratinocytes and leukocytes	EVER proteins may be involved in the regulation of cellular zinc homeostasis in lymphocytes	HPV (group B1) infections and cancer of the skin (typical EV)	226400
6. Predisposition to severe viral infection						
(a) STAT2 deficiency^a^	Mutations of *STAT2*	AR	T and NK cells	STAT2-dependent	Severe viral infections (disseminated vaccine-strain measles)	Not assigned
				IFN-α and -β response	
(b) MCM4 deficiency^a^	Mutations in *MCM4*	AR	NK cells	DNA repair disorder	Viral infections (EBV, HSV, VZV)	609981
					Adrenal failure	
					Short stature	
7. Herpes simplex encephalitis (HSE)						
(a) TLR3 deficiency^a^	(b) Mutations of *TLR3*	AD	Central nervous system (CNS) resident cells and fibroblasts	TLR3-dependent	Herpes simplex virus 1 encephalitis (incomplete clinical penetrance for all etiologies listed here)	613002
		AR		IFN-α, -β, and -λ induction	
(b) UNC93B1 deficiency^a^	(a) Mutations of *UNC93B1*	AR	CNS resident cells and fibroblasts	UNC-93B-dependent	Herpes simplex virus 1 encephalitis	610551
				IFN-α, -β, and -λ induction	
(c) TRAF3 deficiency^a^	(c) Mutations of *TRAF3*	AD	CNS resident cells and fibroblasts	TRAF3-dependent	Herpes simplex virus 1 encephalitis	614849
				IFN-α, -β, and -λ induction	
(d) TRIF deficiency^a^	(c) Mutations of *TRIF*	AD	CNS resident cells and fibroblasts	TRIF-dependent	Herpes simplex virus 1 encephalitis	614850
		AR		IFN-α, -β, and -λ induction	
(e) TBK1 deficiency^a^	(c) Mutations of *TBK1*	AD	CNS resident cells and fibroblasts	TBK1-dependent	Herpes simplex virus 1 encephalitis	Not assigned
				IFN-α, -β, and -λ induction	
8. Predisposition to invasive fungal diseases^a^						
CARD9 deficiency	Mutations of *CARD9*	AR	Mononuclear phagocytes	CARD9 signaling pathway	Invasive candidiasis infection Deep dermatophytoses	212050
9. Chronic mucocutaneous candidiasis (CMC)						
(a) IL-17RA deficiency^a^	(a) Mutations in *IL-17RA*	AR	Epithelial cells, fibroblasts, mononuclear phagocytes	IL-17RA signaling pathway	CMC Folliculitis	605461
(b) IL-17F deficiency^a^	(b) Mutations in *IL-17F*	AD	T cells	IL-17F-containing dimers	CMC Folliculitis	606496
(c) STAT1 gain-of-function	(c) Gain-of-function mutations in *STAT1*	AD	T cells	Gain-of-function STAT1 mutations that impair the development of IL-17-producing T cells	CMC	614162
					Various fungal, bacterial, and viral (HSV) infections	
					Autoimmunity (thyroiditis, diabetes, cytopenia)	
					Enteropathy	
(d) ACT1 deficiency^a^	(c) Mutations in *ACT1*	AR	T cells, fibroblasts	Fibroblasts fail to respond to IL-17A and IL-17F, and their T cells to IL-17E	CMC	615527
					Blepharitis, folliculitis, and macroglossia	
10. Trypanosomiasis^a^	Mutations in *APOL-I*	AD		APOL-I	Trypanosomiasis	603743
11. Isolated congenital asplenia (ICA)	Mutations in *RPSA*	AD	Spleen	RPSA encodes ribosomal protein SA, a component of the small subunit of the ribosome	Bacteremia (encapsulated bacteria) No spleen	271400

*XL, X-linked inheritance; AR, autosomal recessive inheritance; AD, autosomal dominant inheritance; NF-κB, nuclear factor kappa B; TIR, Toll and interleukin 1 receptor; IFN, interferon; HVP, human papilloma virus; TLR, Toll-like receptor; IL, interleukin*.

*^a^Ten or fewer unrelated cases reported in the literature*.

*Eight new disorders have been added to Table 6. Three new entries have been added in the table. One is a new PID with the association of recurrent bacterial infections, autoinflammation, and amylopectinosis caused by AR HOIL1 mutations found in two kindreds. The second is severe viral infection, for which three genetic etiologies have been discovered. AR-STAT2 deficiency and AR-CD16 deficiency have been found in one kindred each. AR MCM4 deficiency has been found in several Irish kindreds. The third is isolated congenital asplenia identified in 18 patients from 8 kindreds*.

*XR-EDA-ID is highly heterogeneous clinically, both in terms of developmental features (some patients display osteopetrosis and lymphedema, in addition to EDA, while others do not display any developmental features) and infectious diseases (some display multiple infections, viral, fungal, and bacterial, while others display a single type of infection). The various OMIM entries correspond to these distinct clinical diseases*.

**Table 7:** **Autoinflammatory disorders**.

Disease	Genetic defect/presumed pathogenesis	Inheritance	Affected cells	Functional defects	Associated features	OMIM number
1. Defects effecting the inflammasome						
(a) Familial Mediterranean fever	Mutations of *MEFV (lead to gain of pyrin function, resulting in inappropriate IL-1β release)*	AR	Mature granulocytes, cytokine-activated monocytes	Decreased production of pyrin permits ASC-induced IL-1 processing and inflammation following subclinical serosal injury; macrophage apoptosis decreased	Recurrent fever, serositis, and inflammation responsive to colchicine. Predisposes to vasculitis and inflammatory bowel disease	249100
(b) Mevalonate kinase deficiency (hyper IgD syndrome)	Mutations of *MVK (lead to a block in the mevalonate pathway). Interleukin-1beta mediates the inflammatory phenotype*	AR		Affecting cholesterol synthesis; pathogenesis of disease is unclear	Periodic fever and leukocytosis with high IgD levels	260920
(c) Muckle–Wells syndrome	Mutations of *CIAS1 (also called PYPAF1 or NALP3) lead to constitutive activation of the NLRP3 inflammasome*	AD	PMNs monocytes	Defect in cryopyrin, involved in leukocyte apoptosis and NF-κB signaling and IL-1 processing	Urticaria, SNHL, amyloidosis	191900
(d) Familial cold autoinflammatory syndrome	Mutations of *CIAS1* (see above) Mutations of *NLRP12*	AD	PMNs, monocytes	Same as above	Non-pruritic urticaria, arthritis, chills, fever, and leukocytosis after cold exposure	120100
5. Neonatal onset multisystem inflammatory disease (NOMID) or chronic infantile neurologic cutaneous and articular syndrome (CINCA)	Mutations of *CIAS1* (see above)	AD	PMNs, chondrocytes	Same as above	Neonatal onset rash, chronic meningitis, and arthropathy with fever and inflammation	607115
2. Non inflammasome-related conditions						
(a) TNF receptor-associated periodic syndrome (TRAPS)	Mutations of *TNFRSF1* (resulting in increased TNF inflammatory signaling)	AD	PMNs, monocytes	Mutations of 55-kDa TNF receptor leading to intracellular receptor retention or diminished soluble cytokine receptor available to bind TNF	Recurrent fever, serositis, rash, and ocular or joint inflammation	142680
(b) Early-onset inflammatory bowel disease	Mutations in *IL-10 (results in increase many proinflammatory cytokines)*	AR	Monocyte/macrophage, activated T cells	IL-10 deficiency leads to increase of TNFγ and other proinflammatory cytokines	Early-onset enterocolitis enteric fistulas, perianal abscesses, chronic folliculitis	124092
(b) Early-onset inflammatory bowel disease	Mutations in *IL-10RA (see above)*	AR	Monocyte/macrophage, activated T cells	Mutation in IL-10 receptor alpha leads to increase of TNFγ and other proinflammatory cytokines	Early-onset enterocolitis enteric fistulas, perianal abscesses, chronic folliculitis	146933
(b) Early-onset inflammatory bowel disease	Mutations in *IL-10RB (see above)*	AR	Monocyte/macrophage, activated T cells	Mutation in IL-10 receptor beta leads to increase of TNFγ and other proinflammatory cytokines	Early-onset enterocolitis enteric fistulas, perianal abscesses, chronic folliculitis	123889
(c) Pyogenic sterile arthritis, pyoderma gangrenosum, acne (PAPA) syndrome	Mutations of *PSTPIP1* (also called C2BP1) (affects both pyrin and protein tyrosine phosphatase to regulate innate and adaptive immune responses)	AD	Hematopoietic tissues, upregulated in activated T cells	Disordered actin reorganization leading to compromised physiologic signaling during inflammatory response	Destructive arthritis, inflammatory skin rash, myositis	604416
(d) Blau syndrome	Mutations of *NOD2* (also called CARD15) (involved in various inflammatory processes)	AD	Monocytes	Mutations in nucleotide binding site of CARD15, possibly disrupting interactions with lipopolysaccharides and NF-κB signaling	Uveitis, granulomatous synovitis, camptodactyly, rash, and cranial neuropathies, 30% develop Crohn’s disease	186580
10. Chronic recurrent multifocal osteomyelitis and congenital dyserythropoietic anemia (Majeed syndrome)^a^	Mutations of *LPIN2* (increased expression of the proinflammatory genes)	AR	Neutrophils, bone marrow cells	Undefined	Chronic recurrent multifocal osteomyelitis, transfusion-dependent anemia, cutaneous inflammatory disorders	609628
11. DIRA (deficiency of the interleukin 1 receptor antagonist)^a^	Mutations of *IL-1RN* (see functional defect)	AR	PMNs, monocytes	Mutations in the IL-1 receptor antagonist allow unopposed action of Interleukin 1	Neonatal onset of sterile multifocal osteomyelitis, periostitis, and pustulosis	612852
12. DITRA – deficiency of IL-36 receptor antagonist	Mutation in *IL-36RN* (see functional defect)	AR	Keratinocyte leukocytes	Mutations in IL-36RN leads to increase IL-8 production	Pustular psoriasis	614204
13. SLC29A3 mutation	Mutation in SLC29A3 (?)	AR	Leukocyte, bone cells	Macrophage activation?	Hyperpigmentation hypertrichosis	602782
14. CAMPS (CARD14 mediated psoriasis)	Mutation in *CARD14* (see functional defect)	AD	Mainly in keratinocyte	Mutations in CARD14 activate the NF-κB pathway and production of IL-8	Psoriasis	173200
15. Cherubism	Mutation in *SH3BP2* (see functional defect)	AD	Stroma cells, bone cells	Hyperactivated macrophage and increased NF-κB	Bone degeneration in jaws	11840
16. CANDLE (chronic atypical neutrophilic dermatitis with lipodystrophy)	Mutation in *PSMB8* (see functional defect)	AD	Keratinocyte, B cell adipose cells	Mutations cause increase IL-6 production	Dystrophy, panniculitis	256040
17. HOIL1 deficiency	Mutation in *HOIL1* (see functional defect)	AR	PMNs, fibroblast	Mutation in *HOIL1* leads to IL-1β dysfunction	Immunodeficiency autoinflammation amylopectinosis	610924
18. PLAID (PLCγ2 associated antibody deficiency and immune dysregulation)	Mutation in *PLCG2* (see functional defect)	AD	B cells, NK, mast cells	Mutations cause activation of IL-1 pathways	Cold urticaria hypogammaglobulinemia	614878

*AR, autosomal recessive inheritance; AD, autosomal dominant inheritance; PMN, polymorphonuclear cells; ASC, apoptosis-associated speck-like protein with a caspase recruitment domain; CARD, caspase recruitment domain; CD2BP1, CD2 binding protein 1; PSTPIP1, proline/serine/threonine phosphatase-interacting protein 1; SNHL, sensorineural hearing loss; CIAS1, cold-induced autoinflammatory syndrome 1*.

*^a^Ten or fewer unrelated cases reported in the literature*.

*Autoinflammatory diseases are clinical disorders marked by abnormally increased inflammation, mediated predominantly by the cells and molecules of the innate immune system, with a significant host predisposition. While the genetic defect of one of the most common autoinflammatory conditions, PFAPA, is not known, recent studies suggest that it is associated with activation of IL-1 pathway and response to IL-1beta antagonists*.

*Muckle–Wells syndrome, familial cold autoinflammatory syndrome and neonatal onset multisystem inflammatory disease (NOMID), which is also called chronic infantile neurologic cutaneous and articular syndrome (CINCA) are caused by similar mutations in CIAS1 mutations. The disease phenotype in any individual appears to depend on modifying effects of other genes and environmental factors*.

**Table 8:** **Complement deficiencies**.

Disease	Genetic defect; presumed pathogenesis	Inheritance	Functional defect	Associated features	OMIM number
1. C1q deficiency	Mutation in *C1QA, C1QB, C1QC*: classical complement pathway components	AR	Absent CH50 hemolytic activity, defective activation of the classical pathway	SLE, infections with encapsulated organisms	120550; 601269; 120575
			Diminished clearance of apoptotic cells	
2. C1r deficiency	Mutation in *C1R*: classical complement pathway component	AR	Absent CH50 hemolytic activity, defective activation of the classical pathway	SLE, infections with encapsulated organisms	216950
3. C1s deficiency	Mutation in *C1S*: classical complement pathway component	AR	Absent CH50 hemolytic activity, defective activation of the classical pathway	SLE, infections with encapsulated organisms	120580
4. C4 deficiency	Mutation in *C4A, C4B*: classical complement pathway components	AR	Absent CH50 hemolytic activity, defective activation of the classical pathway, defective humoral immune response to carbohydrate antigens in some patients	SLE, infections with encapsulated organisms	120810; 120820
5. C2 deficiency	Mutation in *C2*: classical complement pathway component	AR	Absent CH50 hemolytic activity, defective activation of the classical pathway	SLE, infections with encapsulated organisms, atherosclerosis	217000
6. C3 deficiency	Mutation in *C3*: central complement component	AR, gain-of-function AD	Absent CH50 and AH50 hemolytic activity defective opsonization	Infections; glomerulonephritis	120700
			Defective humoral immune response	Atypical hemolytic–uremic syndrome with gain-of-function mutations	
7. C5 deficiency	Mutation in *C5*: terminal complement component	AR	Absent CH50 and AH50 hemolytic activity; defective bactericidal activity	Neisserial infections	120900
8. C6 deficiency	Mutation in *C6*: terminal complement component	AR	Absent CH50 and AH50 hemolytic activity; defective bactericidal activity	Neisserial infections	217050
9. C7 deficiency	Mutation in *C7*: terminal complement component	AR	Absent CH50 and AH50 hemolytic activity; defective bactericidal activity	Neisserial infections	217070
10. C8 α–γ deficiency	Mutation in *C8A, C8G*: terminal complement components	AR	Absent CH50 and AH50 hemolytic activity; defective bactericidal activity	Neisserial infections	120950
11. C8b deficiency	Mutation in *C8B*: Terminal complement component	AR	Absent CH50 and AH50 hemolytic activity; defective bactericidal activity	Neisserial infections	120960
12. C9 deficiency	Mutation in *C9*: Terminal complement component	AR	Reduced CH50 and AP50 hemolytic activity; deficient bactericidal activity	Mild susceptibility to Neisserial infections	613825
13. C1 inhibitor deficiency	Mutation in *SERPING1*: regulation of kinins and complement activation	AD	Spontaneous activation of the complement pathway with consumption of C4/C2	Hereditary angioedema	138470
			Spontaneous activation of the contact system with generation of bradykinin from high molecular weight kininogen	
14. Factor B^a^	Mutation in *CFB*: activation of the alternative pathway	AD	Gain-of-function mutation with increased spontaneous AH50	aHUS	138470
15. Factor D deficiency	Mutation in *CFD*: regulation of the alternative complement pathway	AR	Absent AH50 hemolytic activity	Neisserial infections	134350
16. Properdin deficiency	Mutation in *CFP*: regulation of the alternative complement pathway	XL	Absent AH50 hemolytic activity	Neisserial infections	312060
17. Factor I deficiency	Mutation in *CFI*: regulation of the alternative complement pathway	AR	Spontaneous activation of the alternative complement pathway with consumption of C3	Infections, Neisserial infections, aHUS, preeclampsia, membranoproliferative glomerulonephritis (MPGN)	610984
18. Factor H deficiency	Mutation in *CFH*: regulation of the alternative complement pathway	AR	Spontaneous activation of the alternative complement pathway with consumption of C3	Infections, Neisserial infections, aHUS, preeclampsia, membranoproliferative glomerulonephritis (MPGN)	609814
19. Factor H-related protein deficiencies	Mutation in *CFHR1-5*: bind C3b	AR	Normal CH50, AH50, autoantibodies to Factor H	aHUS	235400
20. Thrombomodulin^a^	Mutation in *THBD*: regulates complement and coagulant activation	AD	Normal CH50, AH50	aHUS	188040
21. MASP1 deficiency	Mutation in *MASP1*: cleaves C2 and activates MASP2	AR	Deficient activation of the lectin activation pathway, cell migration	Infections, 3MC syndrome	600521
22. MASP2 deficiency^a^	*MASP2*: cleavage of C2 and C4	AR	Deficient activation of the lectin activation pathway	Pyogenic infections; inflammatory lung disease, autoimmunity	605102
23. 3MC syndrome COLEC11 deficiency^a^	Mutation in *COLEC11*: binds MASP1, MASP3	AR	Loss of neural crest cell migration signals	A developmental syndrome of facial dysmorphism, cleft lip and/or palate, craniosynostosis, learning disability, and genital, limb, and vesicorenal anomalies (3MC syndrome)	612502
24. Complement receptor 2 (CR2) deficiency^a^	Mutation in *CD21*	AR	See CD21 deficiency in Table 3		120650
25. Complement receptor 3 (CR3) deficiency	Mutation in *ITGB2*	AR	See LAD1 in Table 5		116920
Membrane cofactor protein (CD46) deficiency	Mutation in *CD46*: dissociates C3b and C4b	AD	Inhibitor of complement alternate pathway, decreased C3b binding	aHUS, infections, preeclampsia	120920
Membrane Attack Complex inhibitor (CD59) deficiency^a^	Mutation in *CD59*: regulates the membrane attack complex formation	AR	Erythrocytes highly susceptible to complement-mediated lysis	Hemolytic anemia, polyneuropathy	107271
Ficolin 3 deficiency^a^	Mutation in *FCN3*: activates the classical complement pathway	AR	Absence of complement activation by the Ficolin 3 pathway	Respiratory infections, abscesses	604973

*XL, X-linked inheritance; AR, autosomal recessive inheritance; AD, autosomal dominant inheritance; MAC, membrane attack complex; SLE, systemic lupus erythematosus; MBP, mannose-binding protein; MASP2, MBP-associated serine protease 2*.

*^a^Ten or fewer unrelated cases reported in the literature*.

*New entities added to Table 8 demonstrate the important role of complement regulators in a group of well-described inflammatory disorders. In particular, we have added mutations in membrane bound as well as surface attached soluble complement regulatory proteins recognized in hemolytic–uremic syndrome, age-related macular degeneration, and preeclampsia. The connecting theme of these otherwise unrelated clinical events is excessive activation or insufficient regulation of C3; these events lead to recruitment of leukocytes and permit secretion of inflammatory and anti-angiogenic mediators that disrupt the vascular bed of the target organ. Alterations in the genes for Factor B (CFB), Factor I (CFI), Factor H (CFH), and CD46 act as susceptibility genes rather than disease causing mutations. Population studies reveal no detectable increase in infections in MBP (also known at mannose-binding lectin – MBL) deficient adults. The 3MC syndrome, a developmental syndrome, has been variously called Carnevale, Mingarelli, Malpuech, and Michels syndrome*.

In this updated version, we have added a new category in Table 9 in which “Phenocopies of PID” are listed. This has resulted from our understanding and study of conditions that present as inherited immunodeficiencies, but which are not due to germline mutations and instead arise from acquired mechanisms. Examples include somatic mutations in specific immune cell populations that give rise to the phenotype of autoimmune lymphoproliferative syndrome (ALPS), and also autoantibodies against specific cytokines or immunological factors, with depletion of these factors leading to immunodeficiency. It is likely that increasing numbers of PID phenocopies will be identified in the future, and this may be the start of a much longer table.

**Table 9:** **Phenocopies of PID**.

Disease	Genetic defect/presumed pathogenesis	Circulating T cells	Circulating B cells	Serum Ig	Associated features/similar PID
**Associated with somatic mutations**
(a) Autoimmune lymphoproliferative syndrome (ALPS–SFAS)	Somatic mutation in *TNFRSF6*	Increased CD4^−^CD8^−^double negative (DN) T alpha/beta cells	Normal, but increased number of CD5^+^ B cells	Normal or increased	Splenomegaly, lymphadenopathy, autoimmune cytopenias
					Defective lymphocyte apoptosis/*ALPS–FAS (=ALPS type Im)*
(b) RAS-associated autoimmune leukoproliferative disease (RALD)	Somatic mutation in *KRAS* (gain-of-function)	Normal	B cell lymphocytosis	Normal or increased	Splenomegaly, lymphadenopathy, autoimmune cytopenias, granulocytosis, monocytosis/*ALPS-like*
(c) RAS-associated autoimmune leukoproliferative disease (RALD)	Somatic mutation in *NRAS* (gain-of-function)	Increased CD4^−^CD8^−^double negative (DN) T alpha/beta cells	Lymphocytosis		Splenomegaly, lymphadenopathy, autoantibodies/*ALPS-like*
**Associated with autoantibodies**
(a) Chronic mucocutaneous candidiasis (isolated or with APECED syndrome)	Germline mutation in *AIRE*AutoAb to IL-17 and/or IL-22	Normal	Normal	Normal	Endocrinopathy, chronic mucocutaneous candidiasis/*CMC*
(b) Adult-onset immunodeficiency	AutoAb to IFN gamma	Decreased naive T cells	Normal	Normal	Mycobacterial, fungal, *Salmonella* VZV infections/*MSMD, or CID*
(c) Recurrent skin infection	AutoAb to IL-6	Normal	Normal	Normal	Staphylococcal infections/*STAT3 deficiency*
(d) Pulmonary alveolar proteinosis	AutoAb to GM-CSF	Normal	Normal	Normal	Pulmonary alveolar proteinosis, cryptococcal meningitis/*CSF2RA deficiency*
(e) Acquired angioedema	AutoAb to CI inhibitor	Normal	Normal	Normal	Angioedema/*C1 INH deficiency* (hereditary angioedema)

As with all complex diseases, any classification cannot be strictly adhered to. Certain conditions fall into more than one category and so appear in more than one table. For example, CD40L ligand deficiency is reported in both Tables 1 and 3 as it was initially identified as a defect of B cell isotype switching but is now known to be a defect of co-stimulatory T cell help and function. Similarly, XLP1 due to defects in SH2D1A is listed in Table 1 – combined immunodeficiencies, due to defects of T cell cytotoxicity, T cell help, and B cell maturation, but also in Table 4 – diseases of immune dysregulation, due to the susceptibility to hemophagocytosis. There is a growing appreciation that there can be wide phenotypic viability within a specific genotype that is a product of varied specific mutations between different patients as well as other host and/or environmental factors. The complexities of these conditions in terms of clinical and immunological presentation and heterogeneity cannot be easily captured in the limited space of a table format. For this reason, the furthest left column contains the Online Mendelian Inheritance in Man (OMIM) reference for each condition to allow access to greater detail and updated information.

The rapid advances in gene identification technology, including the widespread use of whole exome and whole genome sequencing, has meant that the ability to identify gene defects in affected families and even single individuals with inherited diseases has grown enormously. In this report, over 30 new gene defects have been added that were identified since the previous classification in November, 2011. These defects can be found in all major groups of PIDs included in this report. In many cases, the mutations are not necessarily in genes formally implicated in immune cell function but are genes involved in essential cell processes. The more detailed analysis and functional consequences of such defects as illustrated by these PIDs will increase our understanding of the interplay between different cellular processes in the development and function of the immune system.

Among the newly identified, gene defects are many that are to date particular to a single pedigree or individual; such defects may prove exceedingly rare, or indeed may not necessarily be found to recur in other individuals. We have marked conditions for which there are 10 or fewer reported individuals with an asterisk, although historically, following the description of the first few cases, additional individuals with a similar PID phenotype and genotype have often been recognized. It is likely that we will uncover many more “personal” or very rare gene defects over time and that the spectrum of PIDs will become increasingly diverse and complex, due to contributions of both environmental exposures and genetic modifiers to each affected individual. The value of this report therefore to capture and catalog the full spectrum at any one time point becomes increasingly important.

The goal of the IUIS Expert Committee on PIDs is to increase awareness, facilitate recognition, and promote optimal treatment for patients with PIDs. In addition to the current report and previous “classification table” publications, the committee has also produced a “Phenotypic Approach for IUIS PID Classification and Diagnosis: Guidelines for Clinicians at the Bedside,” which aims to lead physicians to particular groups of PIDs starting from clinical features and combining routine immunological investigations. Together, these contributions will hopefully allow a practical clinical framework for PID diagnosis. The committee also aims to establish a classification of PIDs based on other aspects and will work on publishing further guidelines in due course.

## Conflict of Interest Statement

The authors declare that the research was conducted in the absence of any commercial or financial relationships that could be construed as a potential conflict of interest.

