# Corrigendum: Primary Immunodeficiency Diseases: An Update on the Classification from the International Union of Immunological Societies Expert Committee for Primary Immunodeficiency

**DOI:** 10.3389/fimmu.2014.00460

**Published:** 2014-09-24

**Authors:** Waleed Al-Herz, Aziz Bousfiha, Jean-Laurent Casanova, Talal Chatila, Mary Ellen Conley, Charlotte Cunningham-Rundles, Amos Etzioni, Jose Luis Franco, H. Bobby Gaspar, Steven M. Holland, Christoph Klein, Shigeaki Nonoyama, Hans D. Ochs, Erik Oksenhendler, Capucine Picard, Jennifer M. Puck, Kate Sullivan, Mimi L. K. Tang

**Affiliations:** ^1^Department of Pediatrics, Kuwait University, Kuwait City, Kuwait; ^2^Allergy and Clinical Immunology Unit, Department of Pediatrics, Al-Sabah Hospital, Kuwait City, Kuwait; ^3^Clinical Immunology Unit, Casablanca Children’s Hospital, Ibn Rochd Medical School, King Hassan II University, Casablanca, Morocco; ^4^St. Giles Laboratory of Human Genetics of Infectious Diseases, Rockefeller Branch, The Rockefeller University, New York, NY, USA; ^5^Laboratory of Human Genetics of Infectious Diseases, Necker Branch, INSERM UMR1163, Imagine Institut, Necker Medical School, University Paris Descartes, Paris, France; ^6^Division of Immunology, Children’s Hospital Boston, Boston, MA, USA; ^7^Department of Medicine and Pediatrics, Mount Sinai School of Medicine, New York, NY, USA; ^8^Meyer Children’s Hospital-Technion, Haifa, Israel; ^9^Group of Primary Immunodeficiencies, University of Antioquia, Medellin, Colombia; ^10^UCL Institute of Child Health, London, UK; ^11^Laboratory of Clinical Infectious Diseases, National Institute of Allergy and Infectious Diseases, Bethesda, MD, USA; ^12^Dr. von Hauner Children’s Hospital, Ludwig-Maximilians-University Munich, Munich, Germany; ^13^Department of Pediatrics, National Defense Medical College, Saitama, Japan; ^14^Department of Pediatrics, Seattle Children’s Research Institute, University of Washington, Seattle, WA, USA; ^15^Department of Clinical Immunology, Hôpital Saint-Louis, Assistance Publique-Hôpitaux de Paris, Paris, France; ^16^Sorbonne Paris Cité, Université Paris Diderot, Paris, France; ^17^Centre d’étude des Déficits Immunitaires (CEDI), Hôpital Necker-Enfants Malades, AP-HP, Paris, France; ^18^Department of Pediatrics, UCSF Benioff Children’s Hospital, University of California San Francisco, San Francisco, CA, USA; ^19^Division of Allergy Immunology, Department of Pediatrics, The Children’s Hospital of Philadelphia, Philadelphia, PA, USA; ^20^Murdoch Childrens Research Institute, Melbourne, VIC, Australia; ^21^Department of Paediatrics, University of Melbourne, Melbourne, VIC, Australia; ^22^Department of Allergy and Immunology, Royal Children’s Hospital, Melbourne, VIC, Australia

**Keywords:** immunodeficiency, classification, gene defects, genotype, IUIS

In Table 1 of the original manuscript, two gene defects were inadvertently omitted. These two defects (Cernunnos and DNA ligase IV deficiency) fall into the category of DNA recombination defects and lead to a radiosensitive form of SCID associated with microcephaly and developmental defects. These two genes have now been added to a revised Table 1.

**Table 1:** **Combined immunodeficiencies**.

Disease	Genetic defect/ presumed pathogenesis	Inheritance	Circulating T cells	Circulating B cells	Serum Ig	Associated features	OMIM number
1. T^−^B^+^ Severe combined immunodeficiency (SCID)							
(a) γc deficiency	Mutation of *IL2RG* Defect in γ chain of receptors for IL-2, -4, -7, -9, -15, -21	XL	Markedly decreased	Normal or increased	Decreased	Markedly decreased NK cells	300400
(b) JAK3 deficiency	Mutation of *JAK3* Defect in Janus activating kinase 3	AR	Markedly decreased	Normal or increased	Decreased	Markedly decreased NK cells;	600173
(c) IL7Rα deficiency	Mutation of *IL7RA* Defect in IL-7 receptor α chain	AR	Markedly decreased	Normal or increased	Decreased	Normal NK cells	146661
(d) CD45 deficiency^a^	Mutation of *PTPRC* Defect in CD45	AR	Markedly decreased	Normal	Decreased	Normal γ/δ T cells	151460
(e) CD3δ deficiency	Mutation of *CD3D* Defect in CD3δ, chain of T cell antigen receptor complex	AR	Markedly decreased	Normal	Decreased	Normal NK cells No γ/δ T cells	186790
(f) CD3ε deficiency^a^	Mutation of *CD3E* Defect in CD3ε chain of T cell antigen receptor complex	AR	Markedly decreased	Normal	Decreased	Normal NK cells No γ/δ T cells	186830
(g) CD3ζ deficiency^a^	Mutation of *CD3Z* Defect in CD3ζ chain of T-cell antigen receptor complex	AR	Markedly decreased	Normal	Decreased	Normal NK cells No γ/δ T cells	186740
(h) Coronin-1A deficiency^a^	Mutation of *CORO1A* Defective thymic egress of T cells and defective T cell locomotion	AR	Markedly decreased	Normal	Decreased	Detectable thymus EBV-associated B-cell lymphoproliferation	605000
2. T^−^B^−^SCID							
(i) DNA recombination defects							
(a) RAG 1 deficiency	Mutation of *RAG1* Defective VDJ recombination Defect of recombinase activating gene (RAG) 1	AR	Markedly decreased	Markedly decreased	Decreased		601457
(a) RAG 2 deficiency	Mutation of *RAG2* Defective VDJ recombination; defect of recombinase activating gene (RAG) 2	AR	Markedly decreased	Markedly decreased	Decreased		601457
(b) DCLRE1C (Artemis) deficiency	Mutation of *ARTEMIS* Defective VDJ recombination; defect in Artemis DNA recombinase-repair protein	AR	Markedly decreased	Markedly decreased	Decreased	Radiation sensitivity	602450
(c) DNA PKcs deficiency^a^	Mutation of *PRKDC* Defective VDJ recombination; defect in DNA PKcs; Recombinase repair protein	AR	Markedly decreased	Markedly decreased	Decreased	Radiation sensitivity, microcephaly, and developmental defects	600899
d) Cernunnos/XLF deficiency	Mutation of *Cernunnos* Defective VDJ recombination; defect in Cernunnos	AR	Markedly decreased	Markedly decreased	Decreased	Radiation sensitivity, microcephaly, and developmental defects	611291
e) DNA ligase IV deficiency	Mutation of *LIG4* Defective VDJ recombination; defect in DNA ligase IV	AR	Markedly decreased	Markedly decreased	Decreased	Radiation sensitivity, microcephaly, and developmental defects	601837
(ii) Reticular dysgenesis, AK2 deficiency	Mutation of *AK2* Defective maturation of lymphoid and myeloid cells (stem cell defect) Defect in mitochondrial adenylate kinase 2.	AR	Markedly decreased	Decreased or normal	Decreased	Granulocytopenia and deafness	103020
(iii) Adenosine deaminase (ADA) deficiency	Mutation of *ADA* Absent ADA activity, elevated lymphotoxic metabolites (dATP, *S*-adenosyl homocysteine)	AR	Absent from birth (null mutations) or progressive decrease	Absent from birth of progressive decrease	Progressive decrease	Decreased NK cells, often with costochondral junction flaring, neurological features, hearing impairment, lung and liver manifestations; partial ADA deficiency may lead to delayed or milder presentation	102700
**Combined immunodeficiencies generally less profound than severe combined immunodeficiency**							
3. CD40 ligand deficiency	Mutation of *CD40LG* Defects in CD40 ligand (CD40L; also called TNFSF5 or CD154) cause defective isotype switching and impaired dendritic cell signaling	XL	Normal; may progressively decrease	sIgM^+^ and sIgD^+^ B cells present, other surface isotype positive B cells absent	IgM increased or normal, other isotypes decreased	Neutropenia, thrombocytopenia; hemolytic anemia, biliary tract and liver disease, opportunistic infections	300386
4. CD40 deficiency^a^	Mutation of *CD40* (also called TNFRSF5); defects in CD40 cause defective isotype switching and impaired dendritic cell signaling	AR	Normal	IgM^+^ and IgD^+^ B cells present, other isotypes absent	IgM increased or normal, other isotypes decreased	Neutropenia, gastrointestinal and liver/biliary tract disease, opportunistic infections	109535
5. Purine nucleoside phosphorylase (PNP) deficiency	Mutation of *PNP* Absent PNP, T cell and neurologic defects from elevated toxic metabolites, especially dGTP	AR	Progressive decrease	Normal	Normal or decreased	Autoimmune hemolytic anemia, neurological impairment	164050
6. CD3γ deficiency^a^	Mutation of *CD3G* Defect in CD3 γ-component of the T cell antigen receptor complex	AR	Normal, but reduced TCR expression	Normal	Normal		186740
7. CD8 deficiency^a^	Mutation of *CD8A* Defects of CD8 α chain – important for maturation and function of CD8 T cells	AR	Absent CD8, normal CD4 cells	Normal	Normal		186910
8. ZAP-70 deficiency	Mutation in ZAP70 intracellular signaling kinase, acts downstream of TCR	AR	Decreased CD8, normal CD4 cells	Normal	Normal	Autoimmunity in some cases	176947
9. MHC class I deficiency	Mutations in *TAP1, TAP2* or *TAPBP* (tapasin) genes giving MHC class I deficiency	AR	Decreased CD8, normal CD4	Normal	Normal	Vasculitis; pyoderma gangrenosum	604571
10. MHC class II deficiency	Mutation in transcription factors for MHC class II proteins (*CIITA, RFX5, RFXAP, RFXANK* genes)	AR	Normal number, decreased CD4 cells	Normal	Normal or decreased	Failure to thrive, diarrhea, respiratory tract infections liver/biliary tract disease	209920
11. ITK deficiency^a^	Mutations in *ITK* encoding IL-2 inducible T cell kinase required for TCR-mediated activation	AR	Progressive decrease	Normal	Normal or decreased	EBV associated B cell lymphoproliferation, lymphoma; normal or decreased IgG	613011
12. SH2D1A deficiency (XLP1)	Mutations in *SH2D1A* encoding an adaptor protein regulating intracellular signals	XL	Normal or Increased activated T cells	Reduced Memory B cells	Partially defective NK cell and CTL cytotoxic activity	Clinical and immunologic features triggered by EBV infection: HLH, lymphoproliferation, Aplastic anemia, lymphoma; hypogammaglobulinemia, absent iNKT cell	308240
13. Cartilage hair hypoplasia	Mutations in *RMRP* (RNase MRP RNA) Involved in processing of mitochondrial RNA and cell cycle control	AR	Varies from severely decreased (SCID) to normal; impaired lymphocyte proliferation	Normal	Normal or reduced. Antibodies variably decreased	Can present just as combined immunodeficiency without other features of short limbed dwarfism, also see Table 2	250250
14. MAGT1 deficiency^a^	Mutations in *MAGT1*, impaired Mg^++^ flux leading to impaired TCR signaling	XL	Decreased CD4 cells reduced numbers of RTE, impaired T-cell proliferation in response to CD3	Normal	Normal	EBV infection, lymphoma; viral infections, respiratory, and GI infections	300715
15. DOCK8 deficiency	Mutations in *DOCK8* – regulator of intracellular actin reorganization	AR	Decreased Impaired T lymphocyte proliferation	Decreased, low CD27+ memory B cells	Low IgM, increased IgE	Low NK cells with impaired function, hypereosinophilia, recurrent infections; severe atopy, extensive cutaneous viral and bacterial (staph.) infections, susceptibility to cancer	243700
16. RhoH Deficiency^a^	Mutations in *RHOH* – an atypical Rho GTPase transducing signals downstream of various membrane receptors	AR	Normal low naïve T cells and RTE, restricted T cell repertoire and impaired T cells proliferation in response to CD3 stimulation	Normal	Normal	HPV infection, lymphoma, lung granulomas, molluscum contagiosum	602037
17. MST1 deficiency	Mutations in *STK4* – a serine/threonine kinase	AR	Decreased increased proportion of terminal differentiated effector memory cells (TEMRA), low naïve T cells, restricted T cell repertoire in the TEMRA population and impaired T cells proliferation	Decreased	High	Recurrent bacterial, viral, and candidal infections; intermittent neutropenia; EBV-driven lymphoproliferation; lymphoma; Congenital heart disease, autoimmune cytopenias; HPV infection	614868
18. TCRα deficiency^a^	Mutations in *TRAC* – essential component of the T cell receptor	AR	Normal All CD3 T cells expressed TCRγδ (or may be better to say: TCRαβ T-cell deficiency), impaired T cells proliferation	Normal	Normal	Recurrent viral, bacterial and fungal infections, immune dysregulation autoimmunity, and diarrhea.	615387
19. LCK deficiency^a^	Defects in *LCK* – a proximal tyrosine kinase that interacts with TCR	AR	Normal total numbers but CD4+ T-cell lymphopenia, low Treg numbers, restricted T cell repertoire and impaired TCR signaling	Normal	Normal IgG and IgA and increased IgM	Diarrhea, recurrent infections, immune dysregulation autoimmunity	153390
20. MALT1 deficiency^a^	Mutations in *MALT1* – a caspase-like cysteine protease that is essential for nuclear factor-kappa-B activation	AR	Normal impaired T cells proliferation	Normal	Normal; impaired antibody response	Bacterial, fungal, and viral infections	604860
21. IL21R deficiency^a^	Defects in *IL21R* – together with common gamma chain binds IL-21	AR	Abnormal T cell cytokine production; Abnormal T cell proliferation to specific stimuli	Normal	Normal but impaired specific responses	Susceptibility to cryptoporidia and pneumocystis and cholangitis	605383
22. UNC119 deficiency^a^	Defects in *UNC119* – an activator of src tyrosine kinases	AD	Low T cells; CD4+ T-cell lymphopenia, impaired TCR signaling	Mostly low	Normal	Recurrent bacterial, fungal, and viral infections	604011
23. CARD11 deficiency^a^	Defects in *CARD11* – acts as a scaffold for NF-κB activity in the adaptive immune response	AR	Normal predominance of naive T-lymphocyte, impaired T cells proliferation	Normal predominance of transitional B lymphocytes	Absent/low	*Pneumocystis jirovicii* pneumonia, bacterial infections	615206
24. OX40 deficiency^a^	Defects in *OX40* – a co stimulatory molecule expressed on activated T cells	AR	Normal T cell numbers; low levels of antigen specific memory CD4+ cells	Normal B cell numbers; lower frequency of memory B cells	Normal	Kaposi’s sarcoma; impaired immunity to HHV8	615593
25. IKBKB deficiency^a^	Defects in *IKBKB* – encodes IκB kinase 2, a component of the NF-κB pathway	AR	Normal total T cells; absent regulatory and γδ T cells; impaired TCR activation	Normal B cell numbers; impaired BCR activation	Decreased	Recurrent bacterial, viral and fungal infections; clinical phenotype of SCID	615592
26. Activated PI3K-δ	Mutation in *PIK3CD*, PI3K-δ	AD gain of function	Decreased total numbers of T cells	Decreased total peripheral B cell and switched memory B cells; increased transitional B cells	Reduced IgG2 and impaired antibody to pneumococci and hemophilus	Respiratory infections, bronchiectasis; autoimmunity; chronic EBV, and CMV infection	602839
27. LRBA deficiency	Mutations in *LRBA* (lipopolysaccharide responsive beige-like anchor protein)	AR	Normal or decreased CD4 numbers; T cell dysregulation	Low or normal numbers of B cells	Reduced I IgG and IgA in most	Recurrent infections, inflammatory bowel disease, autoimmunity; EBV infections	606453
28. CD27 deficiency^a^	Mutations in *CD27*, encoding TNF-R member superfamily required for generation and long-term maintenance of T cell immunity	AR	Normal	No memory B cells	Hypogamma- globulinemia following EBV infection	Clinical and immunologic features triggered by EBV infection, HLH; aplastic anemia, lymphoma, hypogammaglobulinemia, Low iNKT cells	615122
29. Omenn syndrome	Hypomorphic mutations in *RAG1, RAG2*, Artemis, *IL7RA, RMRP, ADA, DNA Ligase IV, IL2RG, AK2*, or associated with DiGeorge syndrome; some cases have no defined gene mutation		Present; restricted T cell repertoire and impaired function	Normal or decreased	Decreased, except increased IgE	Erythroderma, eosinophilia, adenopathies, hepatosplenomegaly	603554

*XL, X-linked inheritance; AR, autosomal recessive inheritance; AD, autosomal dominant inheritance; SCID, severe combined immune deficiencies; EBV, Epstein–Barr virus; Ca^++^, calcium; MHC, major histocompatibility complex, RTE, recent thymic emigrants, HPV, human papillomavirus*.

*^a^Ten or fewer unrelated cases reported in the literature*.

*Infants with SCID who have maternal T cells engraftment may have T cells that do not function normally; these cells may cause autoimmune cytopenias or graft versus host disease. Hypomorphic mutations in several of the genes that cause SCID may result in Omenn syndrome (OS), or “leaky” SCID or a less profound CID phenotype. Both OS and leaky SCID can be associated with higher numbers of T cells and reduced rather than absent activation responses when compared with typical SCID caused by null mutations. A spectrum of clinical findings including typical SCID, OS, leaky SCID, granulomas with T lymphopenia, autoimmunity, and CD4+ T lymphopenia can be found with RAG gene defects. RAC2 deficiency is a disorder of leukocyte motility and is reported in Table 5; however, one patient with RAC2 deficiency was found to have absent T cell receptor excision circles (TRECs) by newborn screening, but T cell numbers and mitogen responses were not impaired. For additional syndromic conditions with T cell lymphopenia, such as DNA repair defects, cartilage hair hypoplasia, IKAROS deficiency, and NEMO syndrome, see Table 2 and 6; however, it should be noted that individuals with the most severe manifestations of these disorders could have clinical signs and symptoms of SCID. Severe folate deficiency (such as with malabsorption due to defects in folate carrier or transporter genes SLC10A1 or PCFT) and some metabolic disorders, such as methylmalonic aciduria, may present with reversible profound lymphopenia in addition to their characteristic presenting features*.

## Conflict of Interest Statement

The authors declare that the research was conducted in the absence of any commercial or financial relationships that could be construed as a potential conflict of interest.

